# Potential of Mixed Dipnictogen Molybdenum Complexes in the Self-Assembly of Thallium Coordination Compounds

**DOI:** 10.1021/acs.inorgchem.4c00867

**Published:** 2024-06-06

**Authors:** Lisa Zimmermann, Christoph Riesinger, Manfred Scheer

**Affiliations:** Institute of Inorganic Chemistry University of Regensburg 93040 Regensburg, Germany

## Abstract

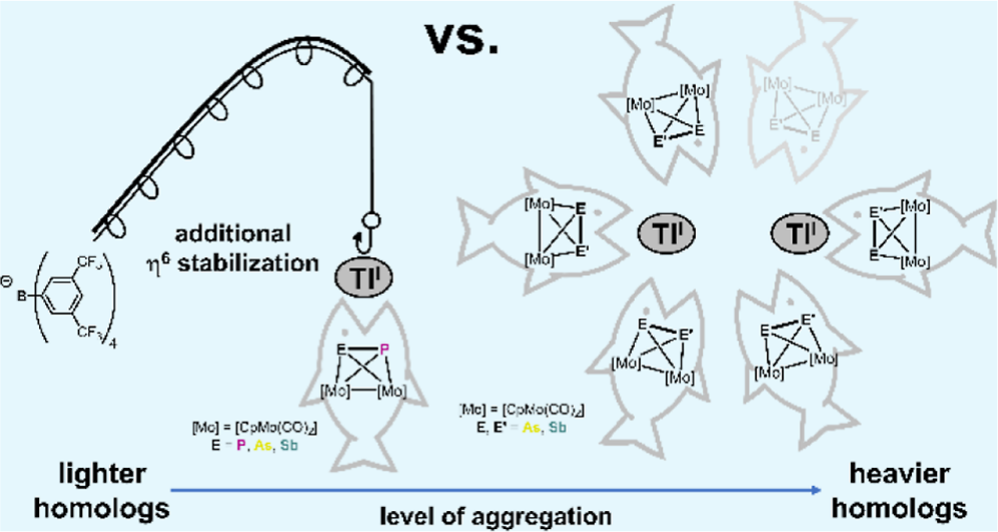

The coordination chemistry of the homo- and heterodipnictogen tetrahedrane complexes [{CpMo(CO)_2_}_2_(μ,η^2:2^-EE′)] (E, E′ = P, As, Sb) (**A–F**) toward Tl[BArF_24_] ([BArF_24_]^−^ = [B(3,5-C_6_H_3_(CF_3_)_2_)_4_]^−^) was studied. Controlled by the used tetrahedranes **A–F**, and thus depending on the respective pnictogen atoms, the monomers [Tl(η^2^-**A**)][BArF_24_] ([**A**]**Tl**) and [Tl(η^2^-**B**)][BArF_24_] ([**B**]**Tl**), the double substituted [Tl(η^1^-**C**)_2_][BArF_24_] ([**C**]_**2**_**Tl**) or the even higher aggregated compounds [Tl_2_(η^2^-**D**)_3_(μ,η^2:1^-**D**)(μ,η^1:1^-**D**)][BArF_24_]_2_ ([**D**]_**5**_**Tl**_**2**_), [Tl_2_(η^2^-**E**)_2_(μ,η^2:1^-**E**)_3_] [BArF_24_]_2_ ([**E**]_**5**_**Tl**_**2**_) and [Tl_2_(η^2^-**F**)_3_(μ,η^2:1^-**F**)_3_][BArF_24_]_2_ ([**F**]_**6**_**Tl**_**2**_) were obtained. Utilization of [BArF_24_]^−^ promises additional stabilization of Tl^I^ via η^6^-coordination of two of its aryl rings as found in compounds [**A**]**Tl**, [**B**]**Tl** and [**C**]_**2**_**Tl**. Within the series of reactivity of **A–F**, the heavier congeners **D**, **E** and **F** tend to form larger aggregates in which σ(E–E′) bond contributions to the coordination behavior were observed. Interatomic distances suggest the presence of Tl···Tl interactions in [**E**]_**5**_**Tl**_**2**_ and [**F**]_**6**_**Tl**_**2**_. The features of the respective coordination compounds were studied in the solid-state as well as in solution. For the latter at least a partial dissociation of the assemblies in solution was indicated. The isolated solid-state aggregates are the first examples of heterodipnictogen units as ligands in self-assembled Tl^I^-based coordination compounds.

## Abstract 2

The reactions of the homo- and hetero dipnictogen tetrahedrane complexes [{CpMo(CO)_2_}_2_(μ,η^2:2^-EE′)] (E, E′ = P, As, Sb) with Tl^I^ salts afford unprecedented coordination compounds. The coordination modes of the EE′ ligands toward the Tl^I^ center(s) differ depending on the used tetrahedrane and allows the formation of unique Tl^I^ dimers.

## Introduction

**Scheme 1:**
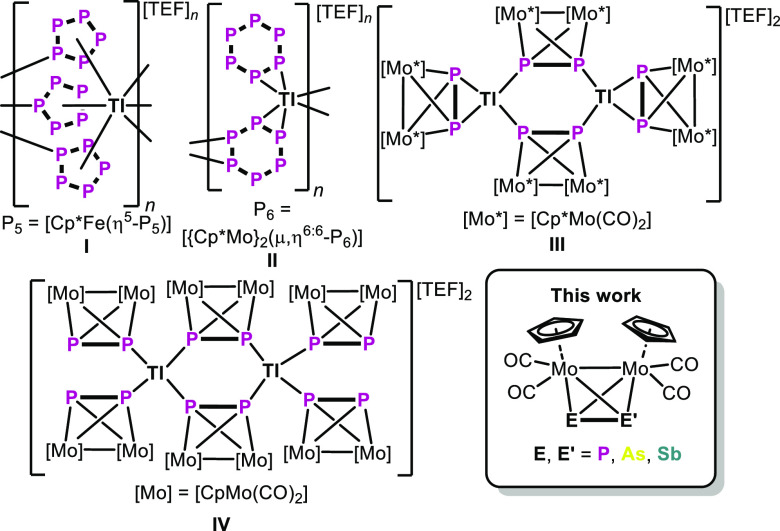
Selected Examples of Tl^I^ Coordination Compounds Based on Reactions of P_*n*_ Ligand Complexes With Tl[TEF], Cp* = C_5_Me_5_, [TEF]^−^ = [Al{OC(CF_3_)_3_}_4_]^−^

Current approaches in supramolecular chemistry focus on the use of nitrogen-, oxygen-, and/or sulfur-containing organic linkers to connect different metal centers.^1−4^ During the last decades, our group has demonstrated that organometallic E_*n*_ (E = heavier group 15 element) ligand complexes are excellent building blocks for supramolecular assemblies as well.^5^ Indeed, many examples of the coordination chemistry of E_*n*_ (*n* = 1–6) ligand complexes toward coinage metal salts, also in combination with organic linkers, were reported during the past two decades.^6,7^ This novel approach allowed the synthesis of a large library of aggregates including polymeric (1D,^8−10^ 2D^11,12^ and 3D^13^ coordination polymers) and discrete (monomeric,^14^ dimeric^15^ or spherical aggregates^16−18^ as well as nanobowls^19^) supramolecular assemblies. While the coinage metal salts of Cu^I^/Ag^I^/Au^I^ have already been extensively utilized in these studies, the monovalent ions of the heavier group 13 elements, namely In^I^ and Tl^I^, have only recently attracted general interest within the field of supramolecular chemistry. This is mostly due to the stability of the +I oxidation state of these ions accompanied by their comparably adaptive coordination behavior. Nevertheless, only little is known, especially about the coordination chemistry of Tl^I^, which is presumably due to it being known to be extremely toxic. The first structurally characterized Tl^I^ arene complex was reported almost 40 years ago by the group of Schmidbaur.^20^ Since then, few examples have been published including Peter’s homoleptic phosphine adduct of Tl^I^,^21^ Power’s Tl_2_[Ar_2_P_4_],^22,23^ Bochmann’s Tl^I^ arene or diethyl ether complexes.^24,25^ Moreover, Mindiola and co-workers prepared PNP pincer complexes of Tl. Very recently, our group studied the coordination chemistry of pnictogenylboranes EH_2_BH_2_·NMe_3_ (E = P, As) toward Tl^I^ salts of different weakly coordinating anions (WCAs).^26^ In terms of polyphosphorus ligand complexes, only few examples are known concerning the coordination chemistry toward thallium metal salts. For instance, the reaction of pentaphosphaferrocene [Cp*Fe(η^5^-P_5_)] (Cp* = C_5_Me_5_) with Tl[TEF] ([TEF]^−^ = [Al{OC(CF_3_)_3_}_4_]^−^) delivered the polymeric compound [Tl{Cp*Fe(η^5:5:1^-P_5_)}_3_]_*n*_[TEF]_*n*_ (Scheme 1, **I**).^27,28^ Moreover, the hexaphosphabenzene complex [{Cp*Mo}_2_(μ,η^6:6^-P_6_)] forms extended 2D networks with Tl[TEF] which can be considered as a supramolecular analog of graphene (Scheme 1, **II**).^29^ Interestingly, the reaction of [{CpMo(CO)_2_}_2_(μ,η^2:2^-P_2_)] (**A**) with Tl[TEF] in CH_2_Cl_2_ delivered the dimeric compound [Tl_2_(η^1^-**A**)_4_(μ,η^1:1^-**A**)_2_][TEF]_2_ consisting of a distorted {Tl_2_P_4_} ring. Each Tl^I^ is further coordinated by two units of **A** in a terminal η^1^ fashion resulting in a distorted tetrahedral geometry of the Tl^I^ centers (Scheme 1, **IV**).^27^ Exchanging the Cp ligand in **A** with a Cp* residue delivers a {Tl_2_P_4_} ring as well. However, each Tl^I^ is now coordinated to only one additional unit of **A**^Cp*^ in an η^2^ fashion, which can be attributed to the higher steric demand of **A**^Cp*^ (Scheme 1, **III**).^30^

The tetrahedrane complexes [{CpMo(CO)_2_}_2_(μ,η^2:2^-EE′)] [E = E′ = P (**A**); E = P, E′ = As (**B**); E = P, E′ = Sb (**C**); E = E′ = As (**D**); E = As, E′ = Sb (**E**); E = E′ = Sb (**F**)], which are isolobal^31^ to the E_4_ (E = P, As) tetrahedranes, are among the simplest in the series of E_*n*_ ligand complexes, as their coordination behavior is more predictable due to only two E-donor atoms. Hence, our group studied the coordination chemistry of the homoleptic congeners [{CpMo(CO)_2_}_2_(μ,η^2:2^-P_2_)] (**A**),^15,32,33^ [{CpMo(CO)_2_}_2_(μ,η^2:2^-As_2_)] (**D**)^34,35^ and [{CpMo(CO)_2_}_2_(μ,η^2:2^-Sb_2_)] (**F**)^36,37^ in detail during the last years. Regarding the more elaborate heterodiatomic complexes [{CpMo(CO)_2_}_2_(μ,η^2:2^-PE)] (E = As (**B**), Sb (**C**)),^38^ only few reports on their coordination toward CuX^39^ (X = Cl, Br, I), (dppf)Cu (dppf = 1,1′-bis(diphenylphosphino)-ferrocene)^40^ and M[TEF]^41^ (M = Ag, Cu) as well as on their oxidation chemistry^42,43^ are known. Their coordination behavior varies due to the different relative energies of the pnictogen lone pairs and the respective σ(E–E′) bonds. Therefore, the question arises as to how the coordination chemistry of the heteroatomic tetrahedranes [{CpMo(CO)_2_}_2_(μ,η^2:2^-EE′)] (E, E′ = P, As, Sb) (**A–F**) toward Tl^I^ salts depends on the used tetrahedrane and thus on the incorporated pnictogen atoms. We herein report on the reactivity of the complexes [{CpMo(CO)_2_}_2_(μ,η^2:2^-EE′)] (E, E′ = P, As, Sb) (**A–F**) toward Tl[BArF_24_] ([BArF_24_]^−^ = [B(3,5-C_6_H_3_(CF_3_)_2_)_4_]^−^) giving first Tl coordination compounds containing mixed heteronuclear group 15 element ligands and unique E/E′ atom-bridged Tl dimers with Tl···Tl interactions.

## Results and Discussion

### Neutral Mononuclear Tl Complexes

**Scheme 2:**
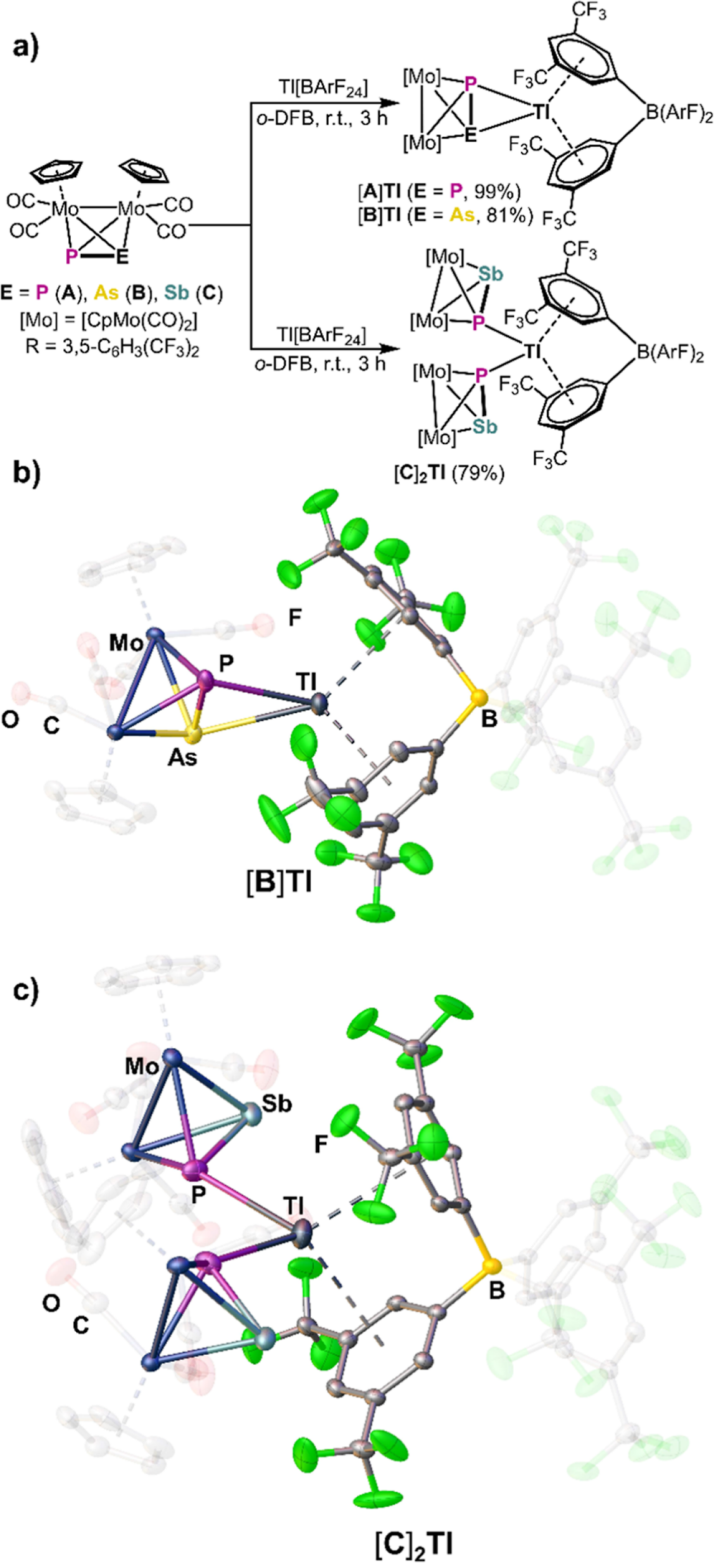
(a) Reaction of A, B and C With Tl[BArF_24_]; Syntheses of [A]Tl, [B]Tl and [C]_2_Tl (Yields are Given in Parentheses),^47^ ArF = 3,5-C_6_H_3_(CF_3_)_2_; (b) Molecular Structure of [B]Tl in the Solid-State; (c) Molecular Structure of [C]_2_Tl in the Solid-State; H Atoms are Omitted for Clarity; Cp and CO Ligands as Well as Parts of [BArF_24_]^−^ are Depicted Translucent

For the following syntheses, [BArF_24_]^−^ ([BArF_24_]^−^ = [B(3,5-C_6_H_3_(CF_3_)_2_)_4_]^−^) was chosen to be used as a weakly coordinating anion (WCA) to provide additional stabilization of the Tl^I^ compounds. Tl[BArF_24_] offers excellent solubility in solvents commonly used (CH_2_Cl_2_, *ortho*-difluorobenzene (*o*-DFB)) in this work while maintaining a suitable crystallization behavior compared to other WCA salts of Tl^I^. In order to ensure similar reaction/crystallization conditions for the used tetrahedranes **A–F**, all reactions were carried out in the same manner: Equimolar amounts of the tetrahedranes **A–F** and Tl[BArF_24_]^44^ were stirred in *o*-DFB at room temperature for 3 h and the reaction mixture was subsequently layered with *n*-hexane for crystallization.^45^ Even if similar reaction conditions were applied, different products were obtained depending on the used tetrahedrane. The reactions of [{CpMo(CO)_2_}_2_(μ,η^2:2^-PE)] (E = P (**A**),^46^ As (**B**)^38^) with Tl[BArF_24_] in *o*-DFB lead to the formation of red blocks of [Tl(η^2^-**A**)][BArF_24_] ([**A**]**Tl**) and [Tl(η^2^-**B**)][BArF_24_] ([**B**]**Tl**), respectively, in excellent crystalline yields^47^ ([**A**]**Tl**: 99%, [**B**]**Tl**: 81%) (Scheme 2a). The formation of higher aggregated compounds as e.g. [Tl_2_(η^1^-**A**)_4_(μ,η^1:1^-**A**)_2_][BArF_24_]_2_ (Scheme 1, **IV**(27)) was not observed, which is attributed to the coordinating abilities of the [BArF_24_]^−^ counterion. Both compounds crystallize in the triclinic space group *P*1̅ and consist of one tetrahedrane which is coordinated to the Tl atom in an η^2^ fashion via the σ(P–E) bond (E = P, As). The Tl^I^ ion is further stabilized by the coordination of two of the aryl rings of the [BArF_24_]^−^ anion in a symmetric η^6^ fashion, which was also observed for Tl[BArF_24_] itself (Scheme 2b).^44^ Indeed, the interpnictogen bonds P–E ([**A**]**Tl** 2.119(2) Å, [**B**]**Tl**: 2.384(2) Å) are slightly elongated compared to the starting materials **A** (2.079(2) Å^46^) and **B** (2.232(2) Å^38^) indicating that the coordination occurs via the σ(P–E) bond. The P–Tl distances in [**A**]**Tl** (P1–Tl: 3.048(1) Å, P2–Tl: 3.208(1) Å) as well as the P–Tl (3.124(1) Å) and As–Tl (3.283(1) Å) distances in [**B**]**Tl** are longer than the P–Tl (2.55 Å^48,49^) or As–Tl (2.65 Å^48,49^) single bonds, but far below the sum of the respective van der Waals radii (P–Tl: 3.76 Å, As–Tl: 3.81 Å).^50^ Interestingly, the heavier homologue [{CpMo(CO)_2_}_2_(μ,η^2:2^-PSb)] (**C**)^38^ reacts under the same reaction conditions to form dark purple block-shaped crystals of [Tl(η^1^-**C**)_2_][BArF_24_] ([**C**]_**2**_**Tl**) in 79% crystalline yield (Scheme 2a).^47^ In contrast to [**A**]**Tl** and [**B**]**Tl**, [**C**]_**2**_**Tl** consists of two units **C** which coordinate to the Tl atom in an η^1^ fashion via the respective lone pair of the phosphorus atoms. The P–Sb bond length (only one bond length is given due to symmetry) in [**C**]_**2**_**Tl** (2.414(2) Å) is indeed slightly shortened compared to **C** (2.470(2) Å^38^) itself, indicating that the coordination occurs almost exclusively via the lone pair of phosphorus (Scheme 2c). The P–Tl distance amounts to 3.083(2) Å and thus is comparable with those observed in **III**(30) or **IV**(27) (Scheme 1). The Sb–Tl distance is 4.127(1) Å and thus larger than the sum of the van der Waals radii (4.02 Å^50^). However, the comparatively small Sb1–P1–Tl angle of 96.56(4) ° indicates some very weak σ(P–Sb)···Tl interactions. Indeed, to the best of our knowledge, compounds [**B**]**T1** and [**C**]**_2_T1** are the first examples of Tl^I^ coordination compounds featuring mixed heavier group 15 ligands. The difference in the coordination behaviors of **A** and **B** compared to **C** toward Tl[BArF_24_] may arise from the higher bond polarity of the P–Sb bond in **C**.

### Electronic Structures of **A–F**

**Figure 1:**
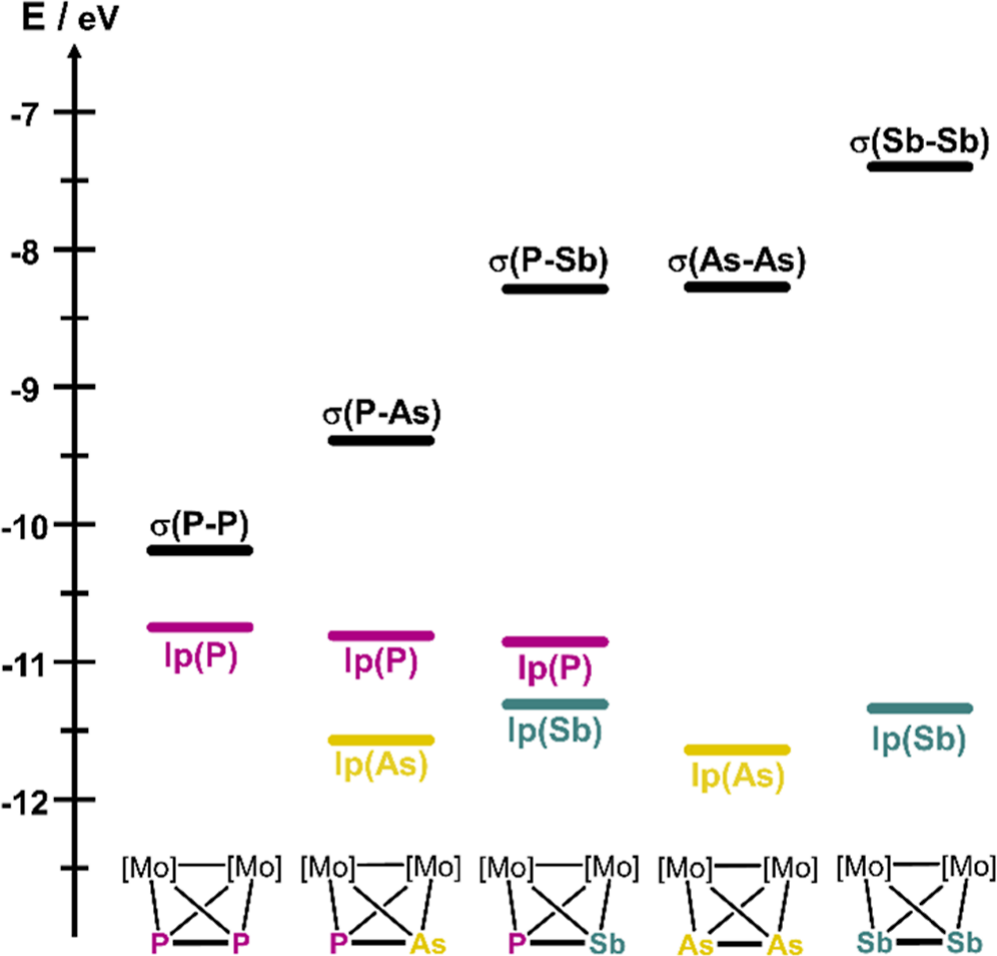
Energy diagram of selected natural bond orbitals for compounds [{CpMo(CO)_2_}_2_(μ,η^2:2^-EE′)] (E, E′ = P, As, Sb); Figure was adapted with permission from ref (35) [Copyright 2024, Wiley-VCH], ref (36) [Copyright 2024, Wiley-VCH] and ref (39) [Copyright 2024, Wiley-VCH].^35,36,39^

The calculated Frontier orbital energy diagrams of **A–C**(39) confirm the lone pairs of As (in **B**) and Sb (in **C**) to be much lower in energy than that of the P atom (in **A**), which is in accordance with the observed coordination via the lone pair of the P atom (Figure 1). Even if the orbital energy of the σ(P–Sb) bond is higher than for σ(P–P) or σ(P–As), no σ(P–Sb) contribution to the Tl bonding is observed for **C** in [**C**]_**2**_**Tl**. Moreover, recently performed natural bond orbital calculations on the tetrahedranes **A**, **D** and **F**(35,36) show that the σ(Sb–Sb) bond in **F** is higher in energy than the σ(As–As) bond in **D** and the σ(P–P) bond in **A**. This indicates clearly that the σ(E–E) bond should be involved in the bonding to the unsaturated Tl^I^ centers (Figure 1).

### Cationic Dinuclear Tl^I^ Complexes

**Scheme 3:**
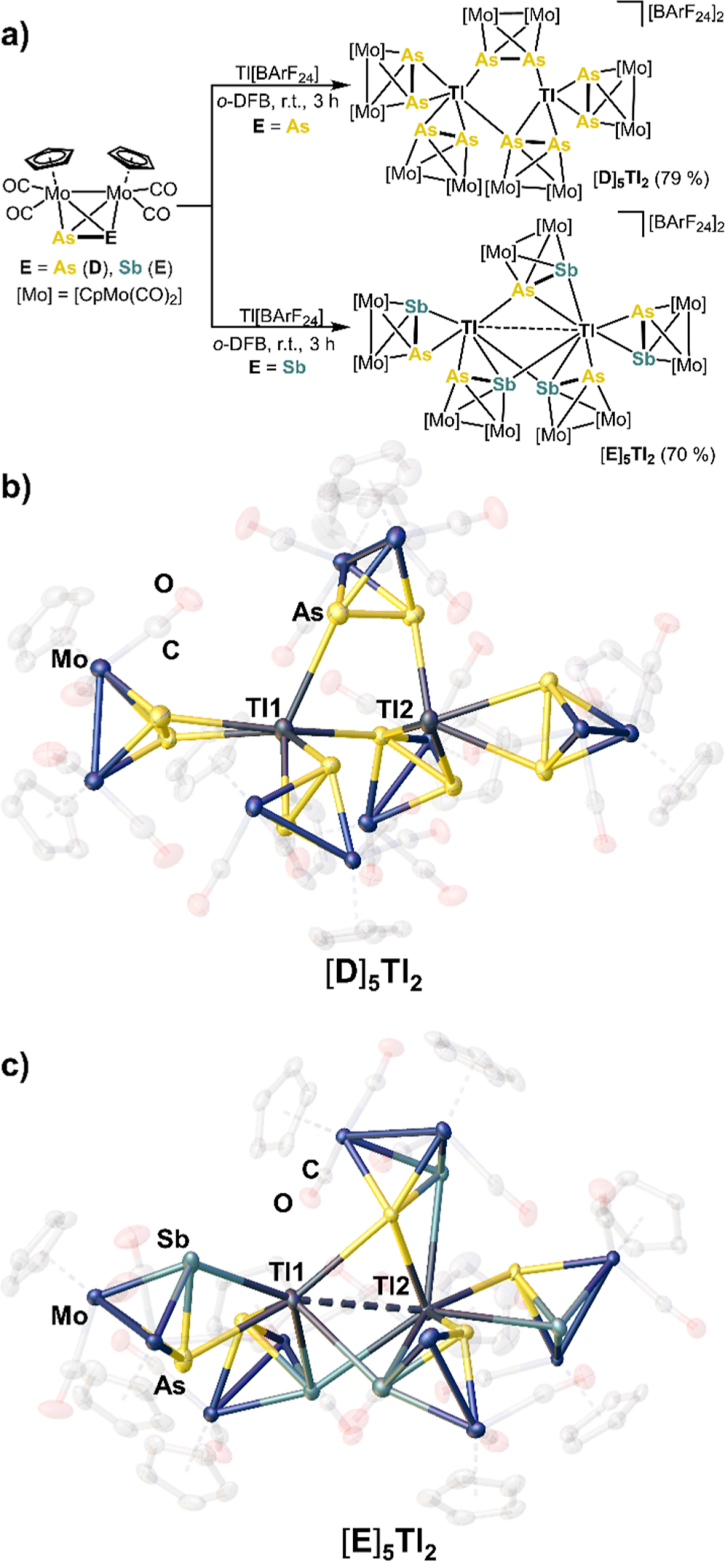
(a) Reaction of D and E With Tl[BArF_24_]; Syntheses of [D]_5_Tl_2_ and [E]_5_Tl_2_ (Yields are Given in Parentheses);^47^ (b) Molecular Structure of the Cationic Part of [D]_5_Tl_2_ in the Solid-State; (c) Molecular Structure of the Cationic Part of [E]_5_Tl_2_ in the Solid State; H Atoms and [BArF_24_]^−^ are Omitted for Clarity; Cp and CO are Depicted Translucent; Ellipsoids are Drawn at the 50% Probability Level

Indeed, reactions of [{CpMo(CO)_2_}_2_(μ,η^2:2^-As_2_)] (**D**)^51^ and [{CpMo(CO)_2_}_2_(μ,η^2:2^-AsSb)] (**E**)^52^ with Tl[BArF_24_] (in a 1:1 ratio) afforded higher aggregated coordination compounds with a 5:2 ratio of **D**/**E** and Tl[BArF_24_], respectively (Scheme 3a), irrespective of the initially used 1:1 stoichiometry.^47^ Surprisingly, the Tl^I^ ion is separated from the [BArF_24_]^−^ ion within these products, which is attributed to the higher donor strength of **D** and **E**.^21,22^ The molecular structure of [Tl_2_(η^2^-**D**)_3_(μ,η^2:1^-**D**)(μ,η^1:1^-**D**)][BArF_24_]_2_ ([**D**]_**5**_**Tl**_**2**_), which crystallizes in form of dark-red plates, reveals a unique Tl^I^ dimer stabilized by five As_2_ ligands of **D** (Scheme 3b). Three ligands **D** show a terminal η^2^-coordination mode, one **D** exhibits a μ,η^2:1^-coordination mode and another ligand **D** shows a bridging μ,η^1:1^-coordination mode. A Tl···Tl interaction is not present due to a Tl1–Tl2 distance of 3.948(2) Å, which is slightly above the sum of their van der Waals radii (3.92 Å).^50^ Thus, Tl1 possesses a distorted tetrahedral geometry, whereas Tl2 shows a trigonal-pyramidal environment (Scheme 3b). The As–As bond lengths (2.331(2) Å-2.342(1) Å) within [**D**]_**5**_**Tl**_**2**_ are, on average, slightly longer compared to the ones in uncoordinated **D** (2.312(3) Å).^51^ The As–Tl interactions are between 3.168(1) Å and 3.545(1) Å, which is fairly long, but still significantly below the sum of their van der Waals radii (3.81 Å).^50^ A comparable coordination mode of the Tl(I) atoms in [**D**]_**5**_**Tl**_**2**_ has not been observed in any other coordination compound of **D**.^34,35^

Although [Tl_2_(η^2^-**E**)_2_(μ,η^2:1^-**E**)_3_][BArF_24_]_2_ ([**E**]_**5**_**Tl**_**2**_) shows a similar composition between **E** and Tl[BArF_24_] as [**D**]_**5**_**Tl**_**2**_, its spatial arrangement in the solid-state differs considerably (Scheme 3c). The core of [**E**]_**5**_**Tl**_**2**_ is composed of two Tl^I^ ions.

However, both show a tetrahedral coordination environment and their distance (Tl1–Tl2:3.882(2) Å) is slightly below the sum of the van der Waals radii (3.92 Å).^50^ Thus, a Tl···Tl interaction appears plausible. The dimer is stabilized by five AsSb ligands of **E** in which two of them each adopt a terminal η^2^-coordination mode while each of the other three units possess a bridging μ,η^2:1^-coordination mode, forming a paddle-wheel-like middle deck (Scheme 3c). As expected from the calculations,^36^ μ,η^2:1^-coordination is favored over a μ,η^1:1^-coordination due to the comparably higher σ(E–E′) orbital energies going from As to Sb. Moreover, two of the three bridging units **E** form a η^1^-coordination via the lone pair of Sb, which is in accordance with the higher energy of the lone pair of Sb compared to the one of As (Figure 1).^36^ All As–Sb bond lengths are slightly elongated [2.531(2)–2.651(3) Å] compared to free **E** (2.515(1) Å).^52^ The As–Tl bond lengths are in the range of 3.148(2) to 3.498(3) Å and the Sb–Tl bond lengths are in between 3.526(2) and 3.874(2) Å, suggesting a partly distorted coordination of the respective σ(As–Sb) bond. Compound [**E**]_**5**_**Tl**_**2**_ is the second example of a self-assembled supramolecular aggregate featuring the heterodipnictogen complex [{CpMo(CO)_2_}_2_(μ,η^2:2^-AsSb)] (**E**) as ligand.^53^

**Scheme 4:**
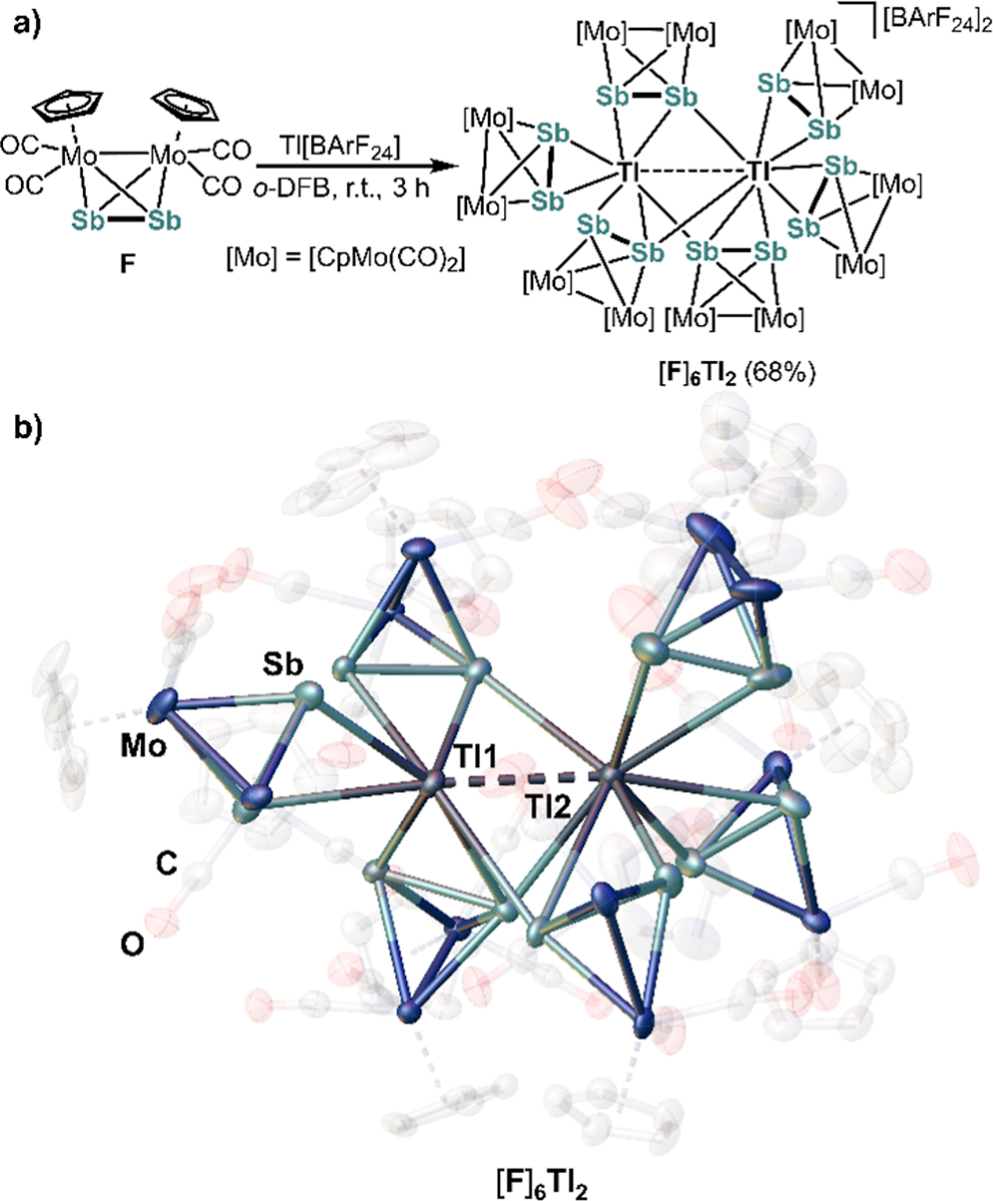
(a) Reaction of F With Tl[BArF_24_]; Synthesis of [F]_6_Tl_2_ (Yield is Given in Parentheses);^47^ (b) Molecular Structure of the Cationic Part of [F]_6_Tl_2_ in the Solid-State; H Atoms and [BArF_24_]^−^ are Omitted for Clarity; Cp and CO Ligands are Depicted Translucent

Furthermore, in order to complete the series of [{CpMo(CO)_2_}_2_(μ,η^2:2^-EE’)] molecules, (E, E’ = P, As, Sb), [{CpMo(CO)_2_}_2_(μ,η^2:2^-Sb_2_)] (**F**)^55^ was reacted with equimolar amounts of Tl[BArF_24_] as well. Interestingly, [Tl_2_(η^2^-**F**)_3_(μ,η^2:1^-**F**)_3_][BArF_24_]_2_ ([**F**]_**6**_**Tl**_**2**_), which was obtained as black blocks upon crystallization from a concentrated solution of [**F**]_**6**_**Tl**_**2**_ in *o*-DFB with *n*-hexane, reveals yet another composition of a MoEE’tetrahedron toward Tl[BArF_24_] (Scheme 4a). [**F**]_**6**_**Tl**_**2**_ consists of six units of **F** coordinated to two Tl^I^ ions. The Tl1–Tl2 distance (3.585(1) Å) is considerably below the sum of the van der Waals radii (3.92 Å)^50^ (Scheme 4b) but significantly longer than expected from the sum of the covalent single-bond radii (2.88 Å).^48,49^ However, it is significantly shorter compared to the ones in [Tl_2_(η^1^-**A**)_4_(μ,η^1:1^-**A**)_2_][TEF]_2_ (5.870(3) Å)^27^ and in [Tl_2_(η^2^-**A**^Cp*^)_2_(μ,η^1:1^-**A**^Cp*^)_2_][TEF]_2_ (5.968(1) Å).^30^ Compared to Power’s dimer Ar′Tl-TlAr′ (Ar′ = C_6_H_3_-2,6-(C_6_H_3_-2,6-Me_2_)_2_, *d*(Tl–Tl) = 3.094(1) Å)^23^ the Tl1–Tl2 distance in [**F**]_**6**_**Tl**_**2**_ is significantly elongated, but in the range of the Tl–Tl contacts observed in Uhl’s tetrameric compound [TlC(SiMe_3_)_3_]_4_ (3.322(1)–3.627(1) Å).^54^ This indicates a possible interaction between the Tl(I) ions, which appears to be more pronounced for the heavier dipnictogen complexes **E** and **F**. [**F**]_**6**_**Tl**_**2**_ contains three Sb_2_ ligands of **F** that adapt a terminal η^2^-coordination mode. The other three Sb_2_ ligands each exhibit a bridging μ,η^2:1^-coordination mode. The Sb–Sb bonds in [**F**]_**6**_**Tl**_**2**_ (2.456(2) Å-2.715(2) Å) are within the range of that found in non-coordinated **F** (2.687(1) Å).^55^ The Sb–Tl bond lengths within [**F**]_**6**_**Tl**_**2**_ are in the range of 3.276(2) Å to 3.585(1) Å, which is fairly long, but still below the sum of their van der Waals radii (4.02 Å).^50^

### Behavior in Solution

Compounds [**A**]**Tl**, [**B**]**Tl**, [**C**]_**2**_**Tl**, [**D**]_**5**_**Tl**_**2**_, [**E**]_**5**_**Tl**_**2**_ and [**F**]_**6**_**Tl**_**2**_ are well soluble in solvents such as CH_2_Cl_2_ or *o*-DFB. All compounds are stable as solids under inert gas for several months. Their ^1^H NMR spectra at room temperature in CD_2_Cl_2_ show a sharp singlet for the Cp ligand in the range of 5.07 ppm to 5.32 ppm as well as characteristic broad signals for the *o*/*p*-H atoms of [BArF_24_]^−^, respectively. The occurrence of only one Cp signal indicates an at least partial dissociation of the assemblies in solution and a fast equilibrium between the formed species in solution. Moreover, the room temperature ^31^P{^1^H} NMR spectra of [**A**]**Tl**, [**B**]**Tl** and [**C**]_**2**_**Tl** exhibit sharp singlets at −31.9 ppm ([**A**]**Tl**), 50.4 ppm ([**B**]**Tl**), and 113.4 ppm ([**C**]_**2**_**Tl**), respectively, and are thus downfield shifted by about 10–20 ppm compared to uncoordinated **A** (−43.7 ppm), **B** (30.1 ppm) and **C** (90.7 ppm). In the ESI mass spectra of all compounds, the most abundant Mo-containing cations are the tetrahedranes [**A–F**]^+^ as well as the species [Tl(**A–F**)]^+^. Furthermore, for the higher aggregated species, also [Tl(**C**–**F**)_2_]^+^ and several decarbonylation products thereof are observed, suggesting that the aggregates remain, at least partially, intact in solution. The ATR-IR spectra of all compounds show *ṽ*(CO) stretches in the range of 1879–1991 cm^–1^ that are comparable with those from the respective starting materials.

## Conclusion

In summary, the potential of the mixed dipnictogen complexes [{CpMo(CO)_2_}_2_(μ,η^2:2^-EE′)] (E, E′ = P, As, Sb) as heterodiatomic donor ligands in the coordination chemistry of Tl^I^ salts was demonstrated. Although a 1:1 ratio of the starting materials was used, the obtained products show different compositions and connectivities depending on the nature of the used tetrahedranes **A–F**. While reactions of [{CpMo(CO)_2_}_2_(μ,η^2:2^-PE)] (E = P (**A**), As (**B**)) with Tl[BArF_24_] yield the monomeric Tl coordination compounds [Tl(η^2^-**A**)][BArF_24_] ([**A**]**Tl**) and [Tl(η^2^-**B**)][BArF_24_] ([**B**]**Tl**), the reaction of [{CpMo(CO)_2_}_2_(μ,η^2:2^-PSb)] (**C**) with Tl[BArF_24_] yields the compound [Tl(η^1^-**C**)_2_][BArF_24_] ([**C**]_**2**_**Tl**). In particular, [**A**]**Tl** and [**B**]**Tl** show π-coordination via the σ(P–E) (E = P, As) bond, whereas [**C**]_**2**_**Tl** exhibits a coordination via the lone pair of the P atom to the Tl center. The Tl centers are stabilized by two of the aryl rings of the counterion [BArF_24_]^−^ in an η^6^-fashion. In contrast, similar reactions performed with [{CpMo(CO)_2_}_2_(μ,η^2:2^-As_2_)] (**D**) and [{CpMo(CO)_2_}_2_(μ,η^2:2^-AsSb)] (**E**) (also in a 1:1 stoichiometry) afforded the higher aggregated coordination compounds [Tl_2_(η^2^-**D**)_3_(μ,η^2:1^-**D**)(μ,η^1:1^-**D**)][BArF_24_]_2_ ([**D**]_**5**_**Tl**_**2**_) and [Tl_2_(η^2^-**E**)_2_(μ,η^2:1^-**E**)_3_][BArF_24_]_2_ ([**E**]_**5**_**Tl**_**2**_) which consist of unique Tl^I^ dimers stabilized by five units of **D** or **E**, respectively. The coordination compound obtained from [{CpMo(CO)_2_}_2_(μ,η^2:2^-Sb_2_)] (**F**) is even higher aggregated and Tl···Tl interactions are suggested for [Tl_2_(η^2^-**F**)_3_(μ,η^2:1^-**F**)_3_][BArF_24_]_2_ ([**F**]_**6**_**Tl**_**2**_) based on the Tl–Tl distances in the solid-state. This indicates that these differences in the reactivity of **A–F** toward Tl^+^ arise from the relative energies of the pnictogen lone pairs and the respective σ(E–E′) bonds. Especially the heavier homologues **D**, **E** and **F** are more susceptible to a σ(E–E′) bond contribution in the coordination to metal centers. Summing up, the obtained complexes discussed within this work reveal unprecedented coordination modes when compared to their intensively studied coinage metal counterparts. These unique structures are only accessible through the utilization of Tl^I^ salts of [BArF_24_]^−^ and open the avenue toward more complex three-dimensional supramolecular aggregates based on simple [{CpMo(CO)_2_}_2_(μ,η^2:2^-EE′)] (E, E′ = P, As, Sb) building blocks.

## Experimental Section

### General Considerations

All manipulations were carried out using standard Schlenk techniques at a Stock apparatus under N_2_ as an inert gas or in a glovebox with Ar atmosphere. The nitrogen inert gas was led over a BASF R 3–1 (CuO/MgSiO_3_) catalyst to remove traces of oxygen. By flowing the nitrogen inert gas through concentrated sulfuric acid, orange gel and sica-pent traces of moisture were eliminated. All glassware was dried with a heatgun (600 °C) for at least 30 min prior to use. *o*-DFB and CD_2_Cl_2_ were distilled from CaH_2_ and other solvents were directly taken from an MBraun SPS-800 solvent purification system and degassed at room temperature prior to use. Solution ^1^H (400.130 MHz), ^11^B (128.379 MHz), ^13^C (100.627 MHz), ^19^F (376.498 MHz) and ^31^P (161.976 MHz) NMR spectra were recorded on a Bruker Avance400 spectrometer using (H_3_C)_4_Si (^1^H, ^13^C), BF_3_·Et_2_O (^11^B), CFCl_3_ (^19^F) or 85% phosphoric acid (^31^P), respectively, as external standards. Chemical shifts (δ) are provided in parts per million (ppm) and coupling constants (*J*) are reported in Hertz (Hz). The following abbreviations are used: s = singlet, d = doublet, dd = doublet of doublets, t = triplet, br = broad and m = multiplet. Elemental analysis of the products was conducted by the elemental analysis department at the University of Regensburg using an Elementar Vario EL. ESI mass spectra were either recorded at the internal mass spectrometry department using a ThermoQuest Finnigan TSQ 7000 mass spectrometer or in our own group using a Micro mass spectrometer. The peak assignment was performed using the molecular weight calculator 6.50.^56^ IR spectra were recorded as solids using a ThermoFisher Nicolet iS5 FT-IR spectrometer with an iD7 ATR module and an ITX Germanium or ITX Diamond crystal. The starting materials [{CpMo(CO)_2_}_2_(μ,η^2:2^-P_2_)] (**A**),^46^ [{CpMo(CO)_2_}_2_(μ,η^2:2^-PAs)] (**B**),^38^ [{CpMo(CO)_2_}_2_(μ,η^2:2^-PSb)] (**C**),^38^ [{CpMo(CO)_2_}_2_ (μ,η^2:2^-As_2_)] (**D**),^51^ [{CpMo(CO)_2_}_2_(μ,η^2:2^-AsSb)] (**E**),^52^ [{CpMo(CO)_2_}_2_(μ,η^2:2^-Sb_2_)] (**F**)^55^ and Tl[BArF_24_]^44^ were synthesized following literature procedures. All other chemicals were purchased from commercial vendors. The thallium compounds used during this work were handled and disposed according to the regulation (EG) Nr. 1272/2008 from the European Union (index number 081-002-00-9; thallium compounds, with the exception of those specified anywhere else in this annex). The following GHS hazard statements need to be considered before using these compounds: H330, H300, H373, H411. Due to their high toxicity, these compounds need to be handled extremely carefully. The respective solids or solutions were disposed in special collecting tanks for Hg-, Tl-, As-, Se-, Be-containing waste.

### Complex Synthesis

All compounds were synthesized for the first time by applying similar reaction conditions: tetrahedranes **A–F** (0.1 mmol) and Tl[BArF_24_] (0.1 mmol) were stirred in *o*-DFB (3 mL) at room temperature for 3 h and subsequently layered with *n*-hexane (30 mL) for crystallization. Since compounds [**A**]**Tl**, [**B**]**Tl**, [**C**]_**2**_**Tl**, [**D**]_**5**_**Tl**_**2**_, [**E**]_**5**_**Tl**_**2**_ and [**F**]_**6**_**Tl**_**2**_ revealed different ratios of **A–F** to Tl[BArF_24_], the experiments were repeated in the stoichiometric ratios obtained from the respective single crystal X-ray experiments. These experiments are described below and revealed the same compounds as in the 1:1 reactions.

### Reaction of [{CpMo(CO)_2_}_2_(μ,η^2:2^-P_2_)] (**A**) with Tl[BArF_24_]

A red solution of [{CpMo(CO)_2_}_2_(μ,η^2:2^-P_2_)] (**A**) (50 mg, 0.1 mmol, 1 equiv) and Tl[BArF_24_] (106 mg, 0.1 mmol, 1 equiv) was stirred in *o*-DFB (3 mL) at room temperature. After 16 h, the mixture was layered with *n*-hexane (20 mL) and red blocks of [Tl(η^2^-**A**)][BArF_24_] ([**A**]**Tl**) suitable for single crystals X-ray analysis were formed after 1 day. The solvent was decanted and the crystals were dried for 2 h at high vacuum (10^–3^ mbar). Crystalline yield: 155 mg (0.099 mmol, 99%). Elemental analysis: calc. (%) for C_46_H_22_O_4_F_24_BP_2_Mo_2_Tl: C: 35.33, H: 1.42. Found (%): C: 35.38, H: 1.35. ESI(+) MS (*o*-DFB): *m*/*z* (%) = 700.79 (100%) [[**A**]**Tl**]^+^ (M^+^), 496.8 (50%) [**A**]^+^, 1196.68 (10%) [2·**A** + Tl]^+^, 246.00 (90%) [Tl(CH_3_CN)]^+^, 204.99 (80%) [Tl]^+^, several decarbonylation products of [**A**]**Tl** and **A** (5–20%). NMR (CD_2_Cl_2_, r.t.): ^**1**^**H**: δ/ppm = 5.18 (s, 10H, C_5_H_5_), 7.45 (br, 4H, *p*-CH [BArF_24_]^−^), 7.61 (br, 8H, *o*-CH [BArF_24_]). ^**31**^**P{**^**1**^**H}**: δ/ppm = −31.9 (s, ω_1/2_ = 9 Hz, 2 P, Mo_2_P_2_–Tl). ^**31**^**P**: δ/ppm = −31.9 (s, 2 P, Mo_2_P_2_–Tl). ^**19**^**F{**^**1**^**H}**: δ/ppm = −62.7 (s, 24 F, *m*-CF_3_). ^**11**^**B{**^**1**^**H}**: δ/ppm = −6.85 (s, 1 B, [BArF_24_]^−^). IR: *ṽ*(CO)/cm^–1^ = 1994 (w), 1937 (m), 1896 (m).

### Reaction of [{CpMo(CO)_2_}_2_(μ,η^2:2^-PAs)] (**B**) with Tl[BArF_24_]

A red solution of [{CpMo(CO)_2_}_2_(μ,η^2:2^-PAs)] (**B**) (54 mg, 0.1 mmol, 1 equiv) and Tl[BArF_24_] (106 mg, 0.1 mmol, 1 equiv) was stirred in *o*-DFB (3 mL) at room temperature. After 16 h, the mixture was layered with *n*-hexane (20 mL) and red blocks of [Tl(η^2^-**B**)][BArF_24_] ([**B**]**Tl**) suitable for single crystals X-ray analysis were formed after 1 day. The solvent was decanted and the crystals were dried for 2 h at high vacuum (10^–3^ mbar). Crystalline yield: 130 mg (0.081 mmol, 81%). Elemental analysis: calc. (%) for C_46_H_22_O_4_F_24_BPAsMo_2_Tl: C: 34.37, H: 1.38. Found (%): C: 34.41, H: 1.22. ESI(+) MS (*o*-DFB): *m*/*z* (%) = 742.77 (40%) [[**B**]**Tl**]^+^ (M^+^), 540.78 (60%) [**B**]^+^, 1284.61 (5%) [2·**B** + Tl]^+^, 246.00 (100%) [Tl(CH_3_CN)]^+^, 204.99 (90%) [Tl]^+^, several decarbonylation products of [**B**]**Tl** and **B** (5–20%). NMR (CD_2_Cl_2_, r.t.): ^**1**^**H**: δ/ppm = 5.15 (s, 10H, C_5_H_5_), 7.45 (br, 4H, *p*-CH [BArF_24_]^−^), 7.61 (br, 8H, *o*-CH [BArF_24_]). ^**31**^**P{**^**1**^**H}**: δ/ppm = 50.4 (s, ω_1/2_ = 14 Hz, 1 P, Mo_2_PAs–Tl). ^**31**^**P**: δ/ppm = 50.4 (s, 1 P, Mo_2_PAs–Tl). ^**19**^**F{**^**1**^**H}**: δ/ppm = −62.7 (s, 24 F, *m*-CF_3_). ^**11**^**B{**^**1**^**H}**: δ/ppm = −6.86 (s, 1 B, [BArF_24_]^−^). IR: *ṽ*(CO)/cm^–1^ = 1991 (w), 1933 (m), 1899 (m).

### Reaction of [{CpMo(CO)_2_}_2_(μ,η^2:2^-PSb)] (**C**) with Tl[BArF_24_]

A red solution of [{CpMo(CO)_2_}_2_(μ,η^2:2^-PSb)] (**C**) (132 mg, 0.24 mmol, 2 equiv) and Tl[BArF_24_] (138 mg, 0.12 mmol, 1 equiv) was stirred in *o*-DFB (3 mL) at room temperature. After 2.5 h, the mixture was layered with *n*-hexane (20 mL) and dark purple blocks of [Tl(η^1^-**C**)_2_][BArF_24_] ([**C**]_**2**_**Tl**) suitable for single crystals X-ray analysis were formed after 1 day. The solvent was decanted and the crystals were dried for 2 h at high vacuum (10^–3^ mbar). Crystalline yield: 212 mg (0.095 mmol, 79%). Elemental analysis: calc. (%) for C_60_H_32_O_8_F_24_BP_2_Sb_2_Mo_4_Tl: C: 32.15, H: 1.44. Found (%): C: 32.31, H: 1.49. ESI(+) MS (*o*-DFB): *m*/*z* (%) = 1378.46 (10%) [[**C**]_**2**_**Tl**]^+^ (M^+^), 790.78 (40%) [**C**–Tl]^+^, 586.78 (100%) [**C**]^+^, 246.00 (90%) [Tl(CH_3_CN)]^+^, 204.99 (90%) [Tl]^+^, several decarbonylation products of [**C**]_**2**_**Tl** and **C** (10%). NMR (CD_2_Cl_2_, r.t.): ^**1**^**H**: δ/ppm = 5.17 (s, 20H, C_5_H_5_), 7.48 (br, 4H, *p*-CH [BArF_24_]^−^), 7.64 (br, 8H, *o*-CH [BArF_24_]). ^**31**^**P{**^**1**^**H}**: δ/ppm = 113.4 (s, ω_1/2_ = 14 Hz, 1 P, Mo_2_PSb–Tl). ^**31**^**P**: δ/ppm = 113.4 (s, 1 P, Mo_2_PSb–Tl). ^**19**^**F{**^**1**^**H}**: δ/ppm = −62.7 (s, 24 F, *m*-CF_3_). ^**11**^**B{**^**1**^**H}**: δ/ppm = −6.85 (s, 1 B, [BArF_24_]^−^). IR: *ṽ*(CO)/cm^–1^ = 1987 (w), 1950 (m), 1908 (s).

### Reaction of [{CpMo(CO)_2_}_2_(μ,η^2:2^-As_2_)] (**D**) with Tl[BArF_24_]

A red solution of [{CpMo(CO)_2_}_2_(μ,η^2:2^-As_2_)] (**D**) (58 mg, 0.10 mmol, 5 equiv) and Tl[BArF_24_] (42 mg, 0.04 mmol, 2 equiv) was stirred in *o*-DFB (3 mL) at room temperature. After 4 h, the mixture was layered with *n*-hexane (20 mL) and red plates of [Tl_2_(η^2^-**D**)_3_(μ,η^2:1^-**D**)(μ,η^1:1^-**D**)][BArF_24_]_2_ ([**D**]_**5**_**Tl**_**2**_) suitable for single crystals X-ray analysis were formed after several days. The solvent was decanted, and the crystals were dried for 2 h at high vacuum (10^–3^ mbar). Crystalline yield: 79 mg (0.016 mmol, 79%). Elemental analysis: calc. (%) for C_134_H_74_O_20_F_48_B_2_As_10_Mo_10_Tl_2_: C: 31.84, H: 1.48. Found (%): C: 32.33, H: 2.16. ESI(+) MS (*o*-DFB): *m*/*z* (%) = 584.70 (50%) [**D** + H]^+^, 788.67 (20%) [**D** + Tl]^+^, 1139.88 (10%) [2·**D**-CO]^+^, 1276.31 (30%) [2·**D** + Tl–CO-Cp]^+^ unidentified, several decarbonylation products (5–10%). NMR (CD_2_Cl_2_, r.t.): ^**1**^**H**: δ/ppm = 5.32 (s, 50H, C_5_H_5_), 7.64 (br, 8H, *p*-CH [BArF_24_]^−^), 7.81 (br, 16H, *o*-CH [BArF_24_]). ^**19**^**F{**^**1**^**H}**: δ/ppm = −62.6 (s, 24 F, *m*-CF_3_). ^**11**^**B{**^**1**^**H}**: δ/ppm = −6.85 (s, 1 B, [BArF_24_]^−^). IR: *ṽ*(CO)/cm^–1^ = 1949 (s), 1896 (s).

### Reaction of [{CpMo(CO)_2_}_2_(μ,η^2:2^-AsSb)] (**E**) with Tl[BArF_24_]

A red solution of [{CpMo(CO)_2_}_2_(μ,η^2:2^-AsSb)] (**E**) (126 mg, 0.20 mmol, 5 equiv) and Tl[BArF_24_] (85 mg, 0.08 mmol, 2 equiv) was stirred in *o*-DFB (3 mL) at room temperature. After 3 h, the mixture was layered with *n*-hexane (20 mL) and dark red plates of [Tl_2_(η^2^-**E**)_2_(μ,η^2:1^-**E**)_3_][BArF_24_]_2_ ([**E**]_**5**_**Tl**_**2**_) suitable for single crystals X-ray analysis were formed after several days. The solvent was decanted, and the crystals were dried for 2 h at high vacuum (10^–3^ mbar). Crystalline yield: 147 mg (0.028 mmol, 70%). Elemental analysis: calc. (%) for C_134_H_74_O_20_F_48_B_2_As_5_Sb_5_Mo_10_Tl_2_: C: 30.43, H: 1.41. Found (%): C: 30.87, H: 1.34. ESI(+) MS (*o*-DFB): *m*/*z* (%) = 1465.96 (5%) [2·**E** + Tl]^+^ (M^+^), 835.17 (10%) [**E**-Tl]^+^, 630.78 (5%) [**E**]^+^, 246.00 (100%) [Tl(CH_3_CN)]^+^, 204.99 (90%) [Tl]^+^, several decarbonylation products (less than 5%). NMR (CD_2_Cl_2_, r.t.): ^**1**^**H**: δ/ppm = 5.22 (s, 20H, C_5_H_5_), 7.56 (br, 4H, *p*-CH [BArF_24_]^−^), 7.73 (br, 8H, *o*-CH [BArF_24_]). ^**19**^**F{**^**1**^**H}**: δ/ppm = −62.7 (s, 24 F, *m*-CF_3_). ^**11**^**B{**^**1**^**H}**: δ/ppm = −6.85 (s, 1 B, [BArF_24_]^−^). IR: *ṽ*(CO)/cm^–1^ = 1955 (m), 1950 (m), 1895 (s).

### Reaction of [{CpMo(CO)_2_}_2_(μ,η^2:2^-Sb_2_)] (**F**) with Tl[BArF_24_]

A dark red solution of [{CpMo(CO)_2_}_2_(μ,η^2:2^-Sb_2_)] (**F**) (68 mg, 0.1 mmol, 6 equiv) and Tl[BArF_24_] (35 mg, 0.033 mmol, 2 equiv) was stirred in *o*-DFB (3 mL) at room temperature. After 2.5 h, the mixture was layered first with *o*-DFB (3 mL) and then with *n*-hexane (20 mL) and black blocks of [Tl_2_(η^2^-**F**)_3_(μ,η^2:1^-**F**)_3_][BArF_24_]_2_ ([**F**]_**6**_**Tl**_**2**_) suitable for single crystals X-ray analysis were formed after 1 week at 4 °C. The solvent was decanted, and the crystals were dried for 2 h at high vacuum (10^–3^ mbar). Crystalline yield: 71 mg (0.090 mmol, 68%). Elemental analysis: calc. (%) for C_148_H_84_O_24_F_48_B_2_Sb_12_Mo_12_Tl_2_·(C_6_H_4_F_2_)_4_: C: 31.03, H: 1.51. Found (%): C: 31.14, H: 1.61. ESI(+) MS (*o*-DFB): *m*/*z* (%) = 1462.24 (100%) [2·**F** + Tl–CO–Cp]^+^, 882.01 (40%) [**F**–Tl]^+^, 677.67 (10%) [**F**]^+^, several decarbonylation products (less than 5%). NMR (CD_2_Cl_2_, r.t.): ^**1**^**H**: δ/ppm = 5.07 (s, 60H, C_5_H_5_), 7.47 (br, 4H, *p*-CH [BArF_24_]^−^), 7.63 (br, 8H, *o*-CH [BArF_24_]). ^**19**^**F{**^**1**^**H}**: δ/ppm = −62.65 (s, 24 F, *m*-CF_3_). ^**11**^**B{**^**1**^**H}**: δ/ppm = −6.85 (s, 1 B, [BArF_24_]^−^). IR: *ṽ*(CO)/cm^–1^ = 1927 (s), 1879 (s).

## Supporting Information Available

NMR spectra, ESI-MS spectra and details on X-ray crystallography and computational studies (PDF)
