# Supplementary material for: Potential of Mixed Dipnictogen Molybdenum Complexes in the Self-Assembly of Thallium Coordination Compounds
Source: Inorg Chem. 2024 Jun 6;63(24):11168–75. doi: 10.1021/acs.inorgchem.4c00867 (PMC11186011; doi:10.1021/acs.inorgchem.4c00867)
Supplement: Supplementary file 1 — ic4c00867_si_001.pdf [file ic4c00867_si_001.pdf]

The Potential of Mixed Dipnictogen Molybdenum Complexes in the Self-Assembly of Thallium Coordination Compounds

Lisa Zimmermann, Christoph Riesinger, Manfred Scheer*

Institute of Inorganic Chemistry
University of Regensburg
93040 Regensburg (Germany)
E-mail: manfred.scheer@chemie.uni-regensburg.de

Contents

1. Author Contributions 3

2. NMR Spectra 4

3. Mass Spectra 8

4. X-ray single crystal structure analysis 12

4.1 General Information 12

4.2 Crystallographic data for the reported structures 13

[Tl(η^2-**A**)][BArF$_{24}$] ([**A**]**Tl**) 13

[Tl(η^2-**B**)][BArF$_{24}$] ([**B**]**Tl**) 14

[Tl(η^1-**C**)$_2$][BArF$_{24}$] ([**C**]$_2$**Tl**) 15

[Tl$_2$(η^2-**D**)$_3$(μ,$\eta^{2:1}$-**D**)(μ,$\eta^{1:1}$-**D**)][BArF$_{24}$]$_2$ ([**D**]$_5$**Tl$_2$**) 16

[Tl$_2$(η^2-**E**)$_2$(μ,$\eta^{2:1}$-**E**)$_3$][BArF$_{24}$]$_2$ ([**E**]$_5$**Tl$_2$**) 17

[Tl$_2$(η^2-**F**)$_3$(μ,$\eta^{2:1}$-**F**)$_3$][BArF$_{24}$]$_2$ ([**F**]$_6$**Tl$_2$**) 18

5. Computational Details 21

6. References 22

1. Author Contributions

Lisa Zimmermann synthesized and characterized all compounds. X-Ray measurements were done by Lisa Zimmermann ([**A**]**Tl**, [**B**]**Tl**, [**C**]$_2$**Tl**, [**D**]$_5$**Tl$_2$**, [**E**]$_5$**Tl$_2$**) and Christoph Riesinger ([**F**]$_6$**Tl$_2$**) and finally checked by Christoph Riesinger. The manuscript (including Figures and Schemes) was written by Lisa Zimmermann. Manfred Scheer supervised the research, raised funding and worked and revised the manuscript.

2. NMR Spectra

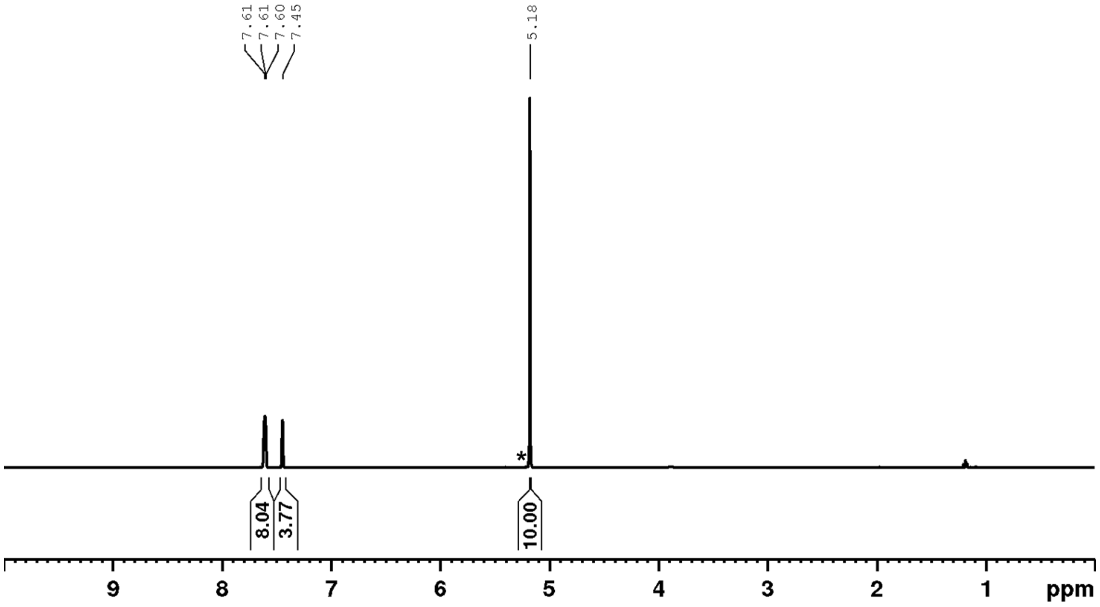

Figure S1: ^{1}H NMR spectrum of [**A**]**Tl** in CD_2Cl_2 at room temperature. *: signal for Cp overlaps with CD_2Cl_2.

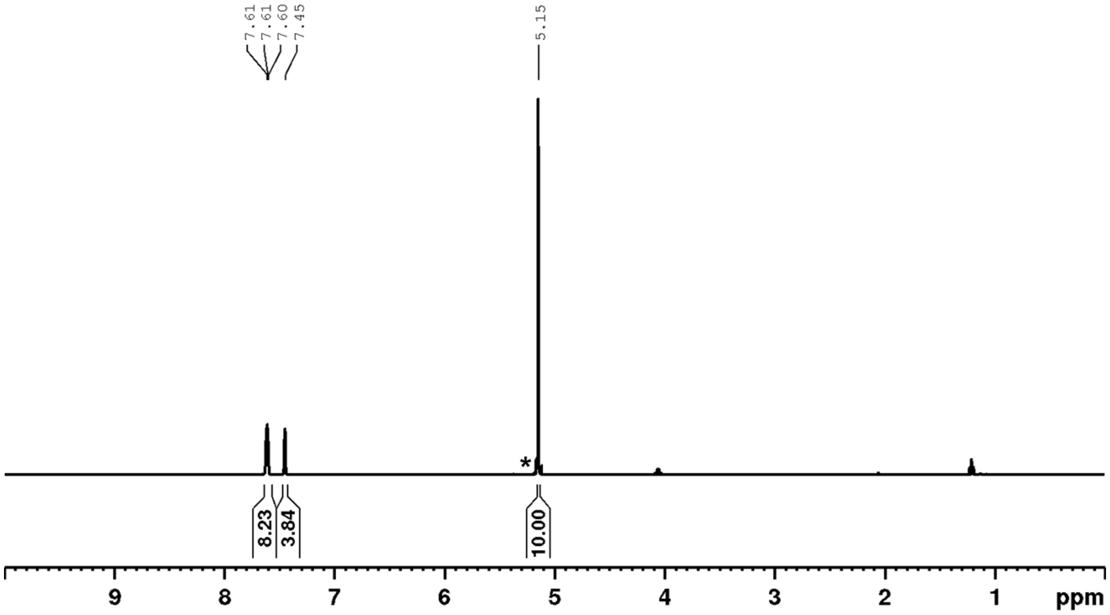

Figure S2: ^{1}H NMR spectrum of [**B**]**Tl** in CD_2Cl_2 at room temperature. *: CD_2Cl_2.

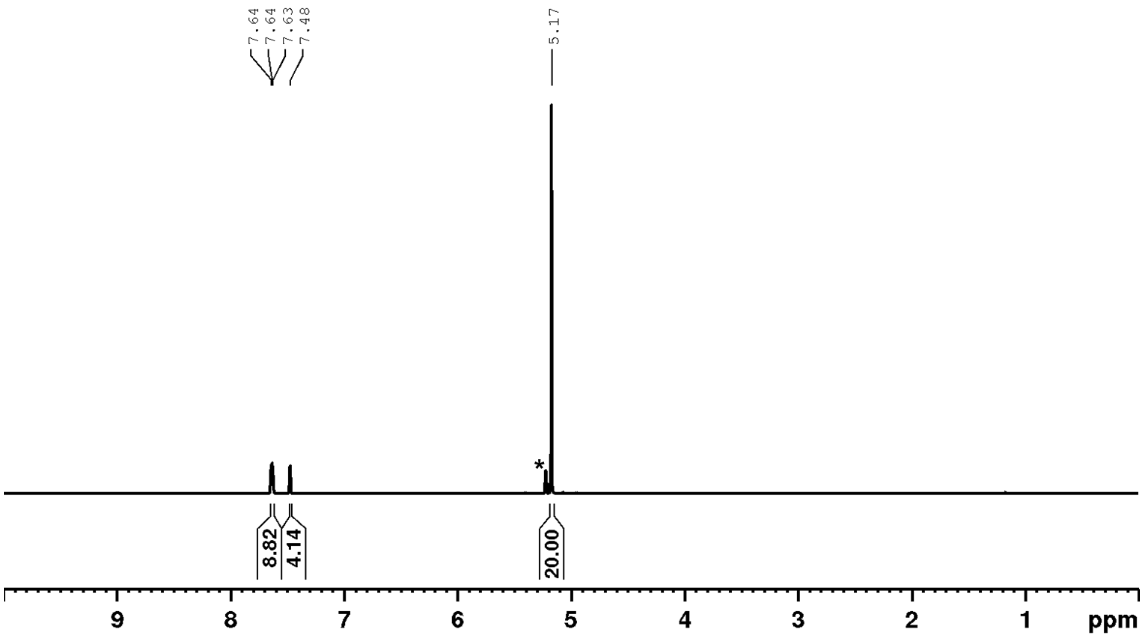

Figure S3: ^{1}H NMR spectrum of [**C**]$_2$**Tl** in CD_2Cl_2 at room temperature. *: CD_2Cl_2.

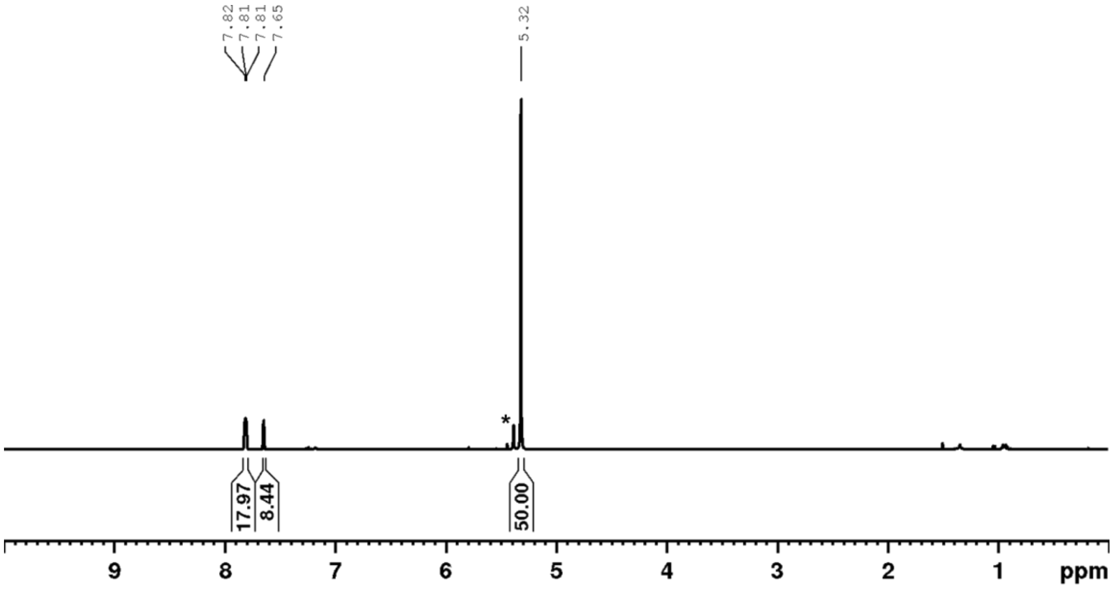

Figure S4: ^{1}H NMR spectrum of [**D**]$_5$**Tl$_2$** in CD_2Cl_2 at room temperature. *: CD_2Cl_2.

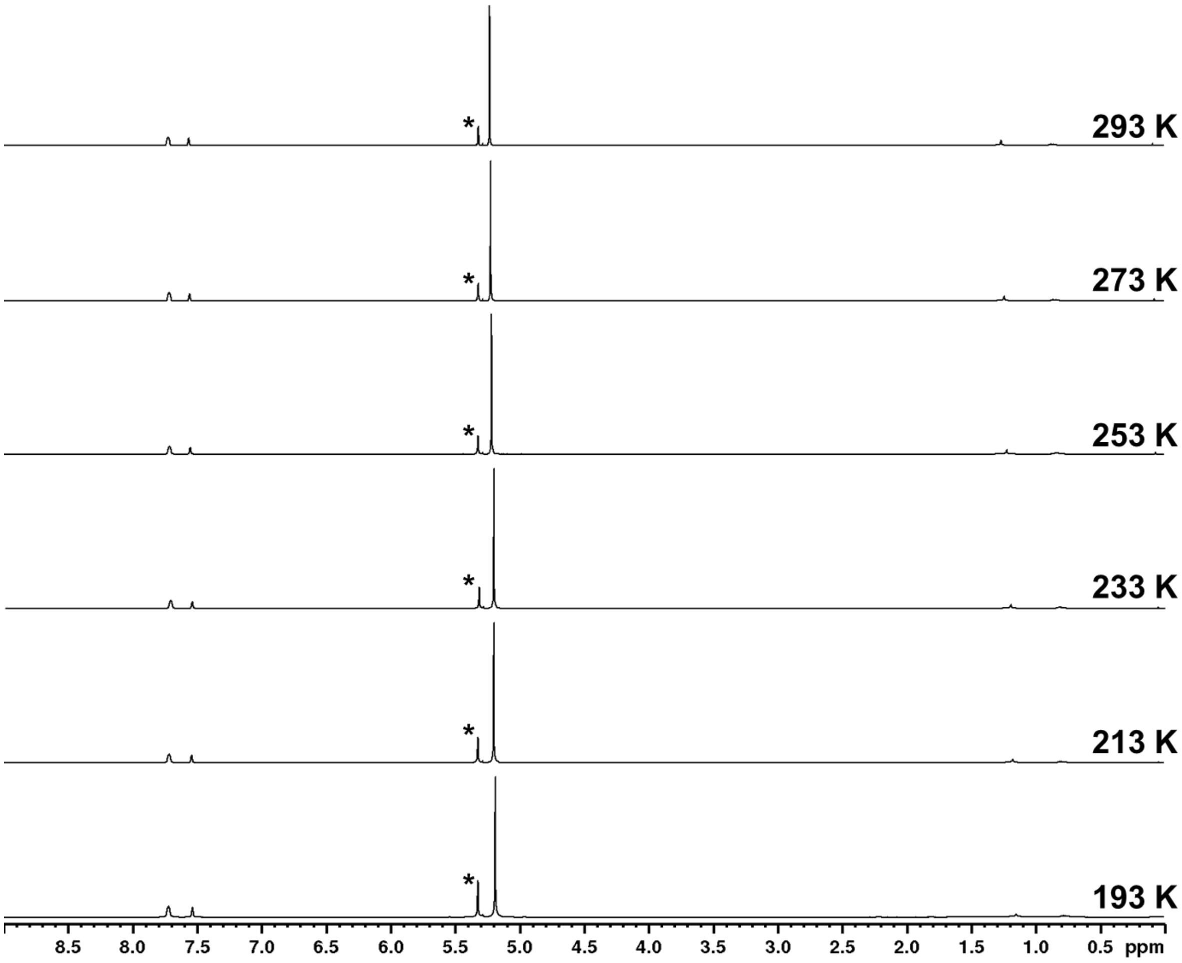

Figure S5: Variable temperature ^{1}H NMR spectrum of [**D**]$_5$**Tl$_2$** in CD_2Cl_2 at room temperature. *: CD_2Cl_2.

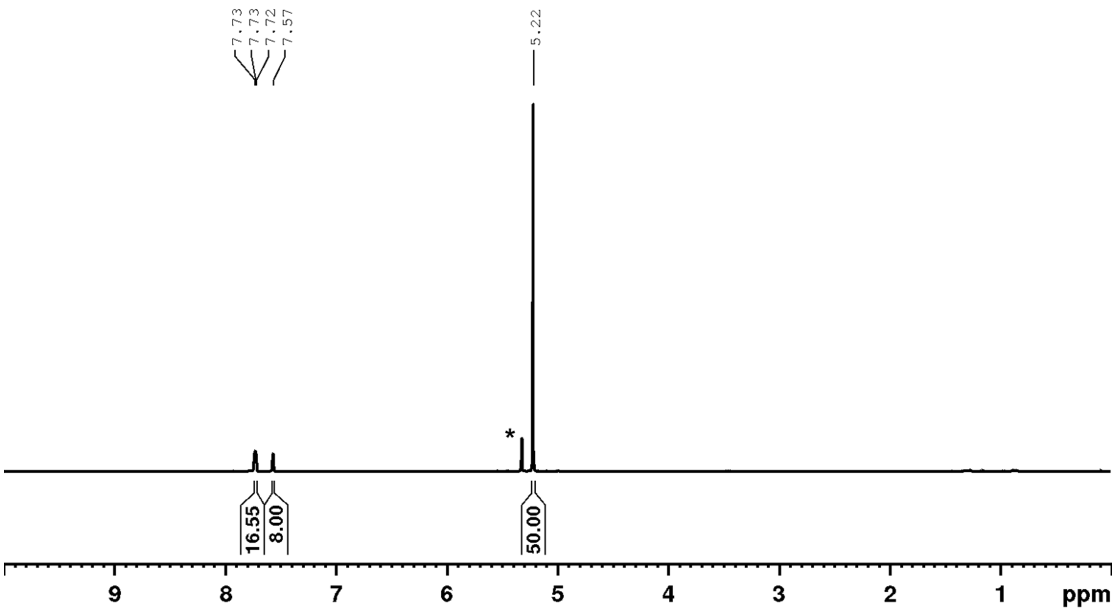

Figure S6: ^{1}H NMR spectrum of [**E**]$_5$**Tl$_2$** in CD_2Cl_2 at room temperature. *: CD_2Cl_2.

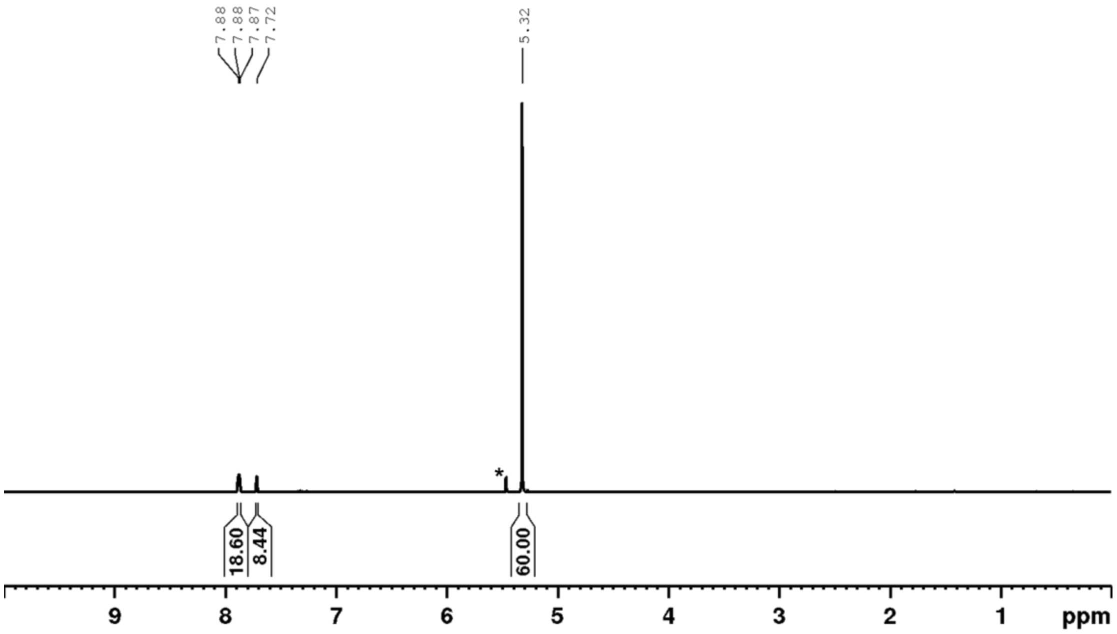

Figure S7: ^{1}H NMR spectrum of [**F**]$_{6}$**Tl**$_{2}$ in CD_2Cl_2 at room temperature. *: CD_2Cl_2.

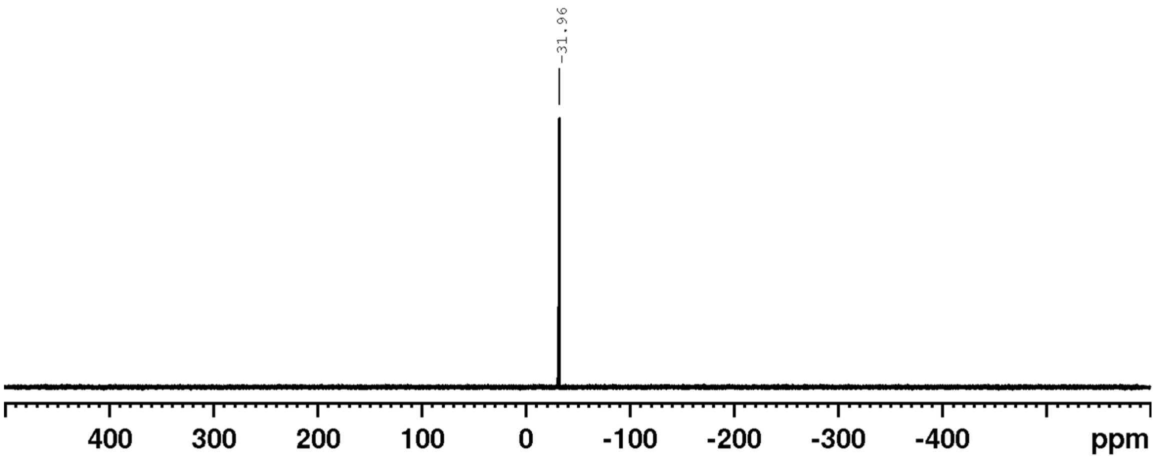

Figure S8: $^{31}P\{^{1}H\}$ NMR spectrum of [**A**]**Tl** in CD_2Cl_2 at room temperature.

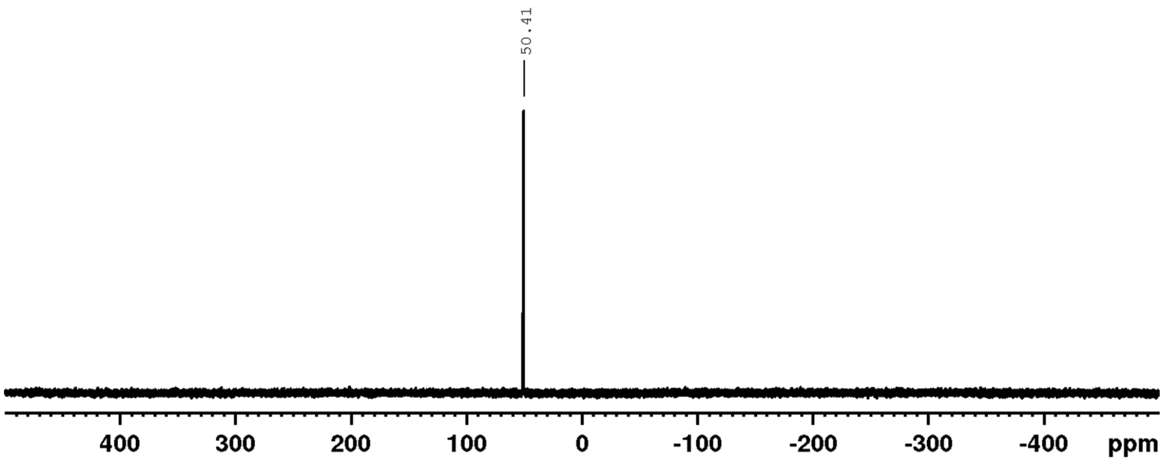

Figure S9: $^{31}P\{^{1}H\}$ NMR spectrum of [**B**]**Tl** in CD_2Cl_2 at room temperature.

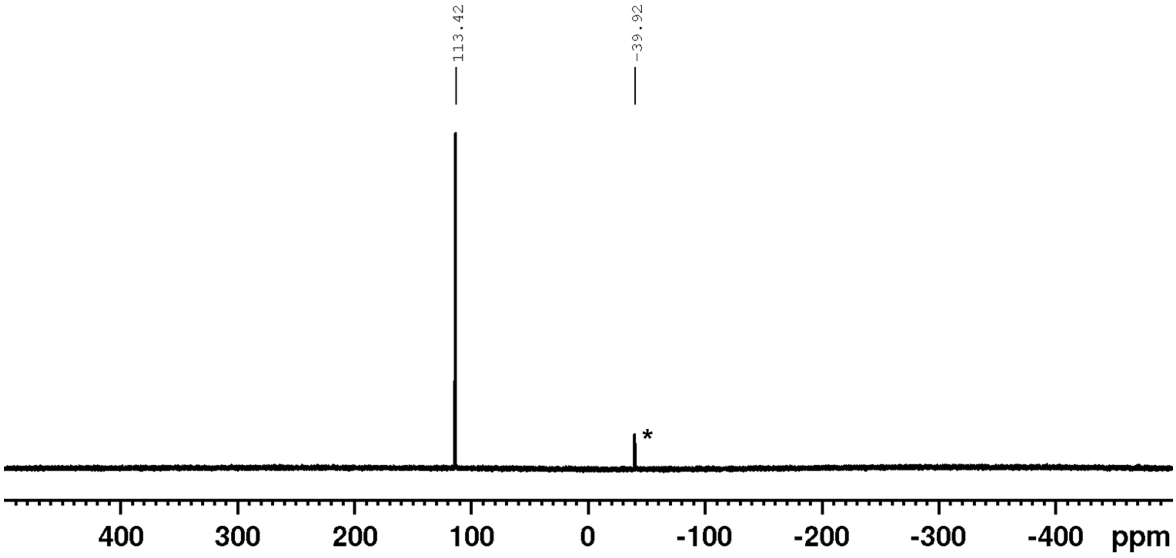

Figure S10: $^{31}P\{^{1}H\}$ NMR spectrum of [**C**]$_2$**Tl** in CD_2Cl_2 at room temperature. *: **A** (4%). The starting material **C** was contaminated with traces of **A**.

3. Mass Spectra

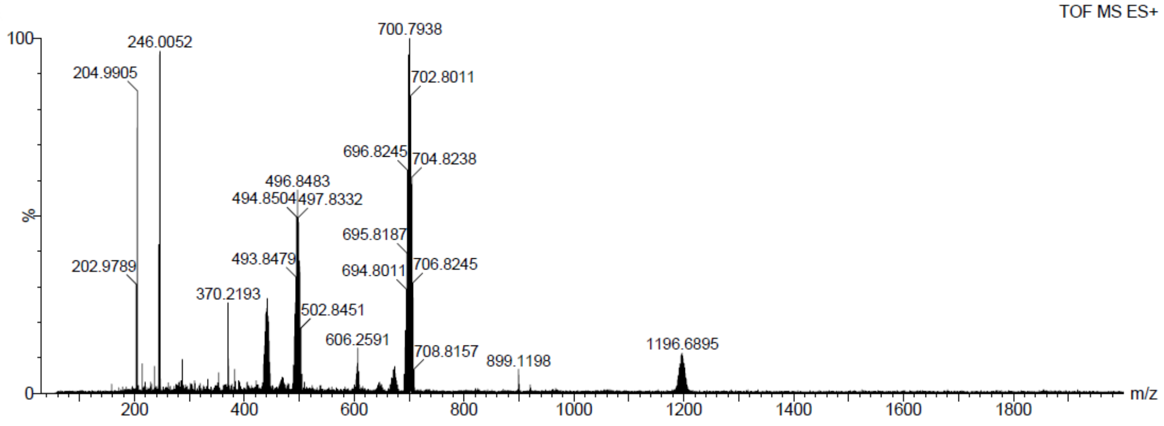

Figure S11: ESI(+) MS spectrum of [**A**]**Tl** in *o*-DFB.

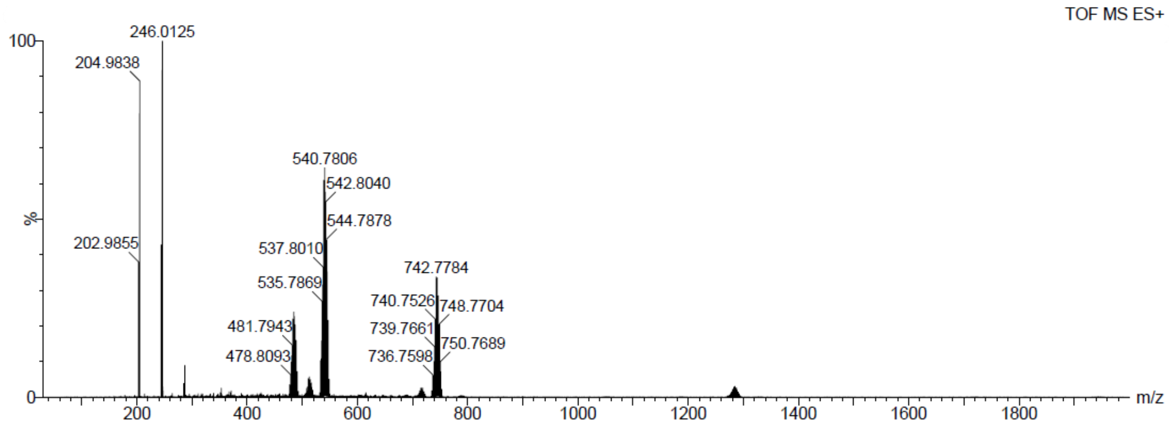

Figure S12: ESI(+) MS spectrum of [**B**]**Tl** in *o*-DFB.

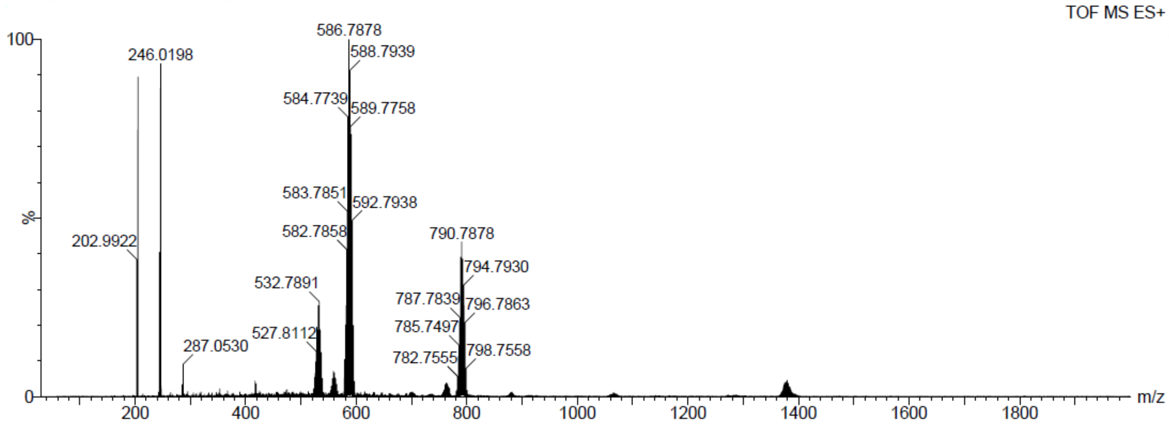

Figure S13: ESI(+) MS spectrum of [**C**]$_2$**Tl** in *o*-DFB.

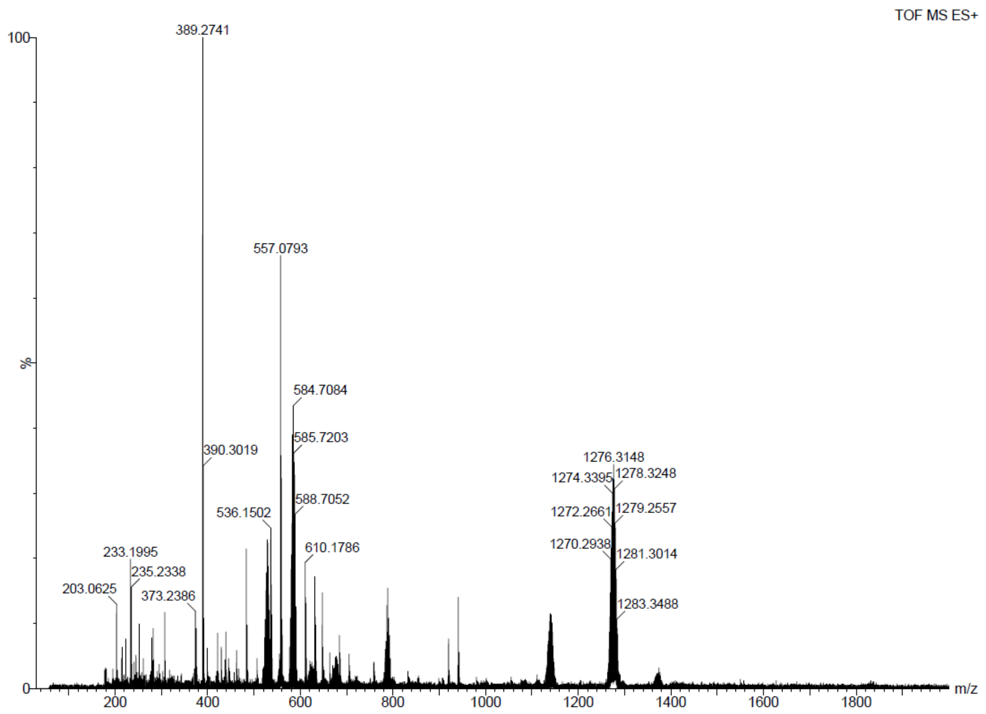

Figure S14: ESI(+) MS spectrum of [**D**]$_5$**TI**$_2$ in *o*-DFB.

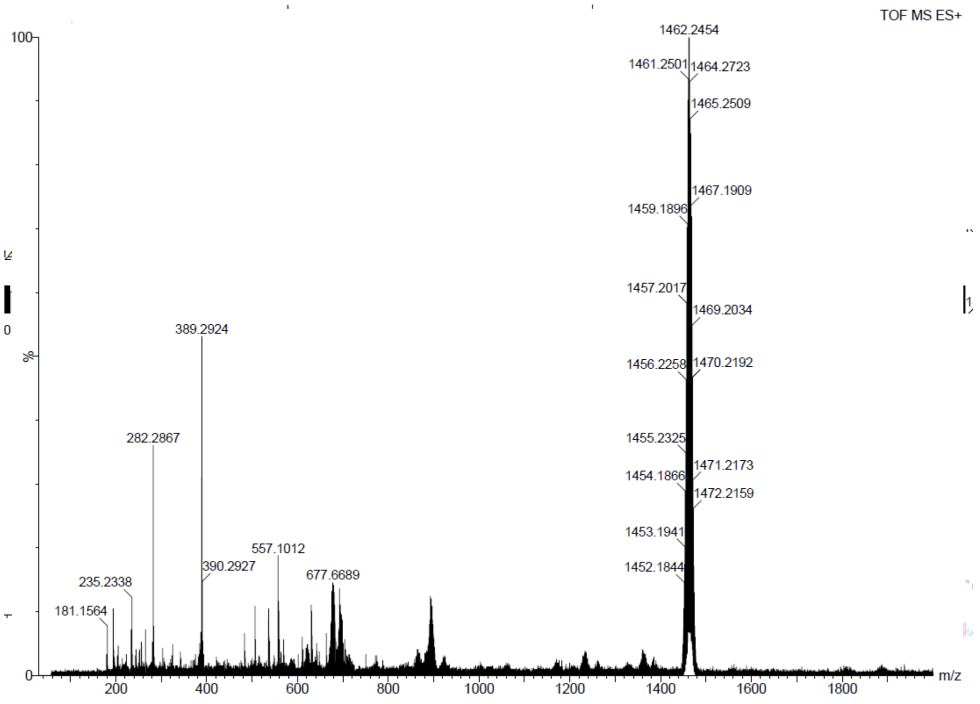

Figure S15: ESI(+) MS spectrum of [**E**]$_5$**TI**$_2$ in *o*-DFB.

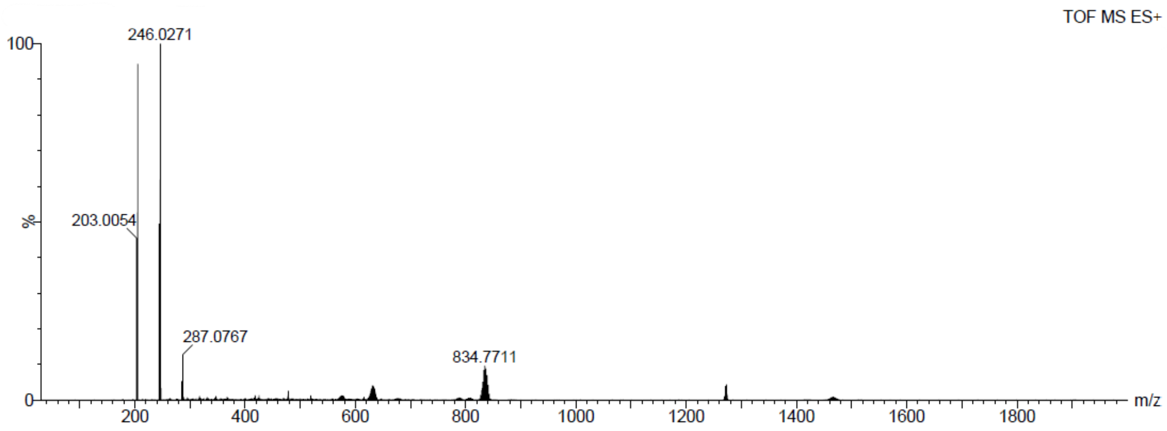

Figure S16: ESI(+) MS spectrum of [**F**]$_6$**Tl$_2$** in *o*-DFB.

4. X-ray single crystal structure analysis

4.1 General Information

The crystallographic data for all synthesized compounds was collected on either a Gemini Ultra diffractometer (Oxford diffraction) with an AtlasS2 detector applying Cu-Kα radiation ([**B**]**Tl**), on a SuperNova diffractometer with a TitanS2 detector using Cu-Kα radiation ([**A**]**Tl**, [**C**]$_2$**Tl**), or on a XtaLAB Synergy R, DW system with HyPix-Arc 150 detector applying Cu-Kα radiation ([**D**]$_5$**Tl$_2$**) or Mo- Kα radiation ([**E**]$_5$**Tl$_2$**, [**F**]$_6$**Tl$_2$**) from a rotating anode X-ray source. All measurements were performed at 123 K. Data collection, data reduction and absorption correction were performed with the CrysAlisPro software package.[1] Structure solution and refinement was conducted in Olex2[2] with ShelXT[3] and ShelXL[4] (full-matric least-squares method against F^2). All non-hydrogen atoms were refined with anisotropic displacement parameters and hydrogen atoms were treated as riding models with isotropic displacement parameters and fixed C-H bond lengths (sp^3: 0.96 (CH_3), 0.97 (CH_2), sp^2: 0.93 (CH)). Visualization of the crystal structures was performed with Olex2.[2]

CCDC-2332190 ([**A**]**Tl**), CCDC-2332191 ([**B**]**Tl**), CCDC-2332192 ([**C**]$_2$**Tl**), CCDC-2332193 ([**D**]$_5$**Tl$_2$**), CCDC-2332194 ([**E**]$_5$**Tl$_2$**), and CCDC-2332195 ([**F**]$_6$**Tl$_2$**), contain the supplementary crystallographic data for this paper. These data can be obtained free of charge at www.ccdc.cam.ac.uk/conts/retrieving.html (or from the Cambridge Crystallographic Data Centre, 12 Union Road, Cambridge CB2 1EZ, UK; Fax: + 44-1223-336-033; e-mail: deposit@ccdc.cam.ac.uk).

4.2 Crystallographic data for the reported structures

[Tl(η^2-**A**)][BArF$_{24}$] ([**A**]**Tl**)

Compound [Tl(η^2-**A**)][BArF$_{24}$] ([**A**]**Tl**) crystallizes from a concentrated solution in *o*-DFB layered with *n*-hexane at room temperature in the triclinic space group $P\bar{1}$ as red blocks. The asymmetric unit contains one molecule **A** and one molecule Tl[BArF$_{24}$]. Disorders within the tetrahedrane unit of **A** and within CF_3 groups of [BArF$_{24}$]$^-$ were treated with adequate restraints.

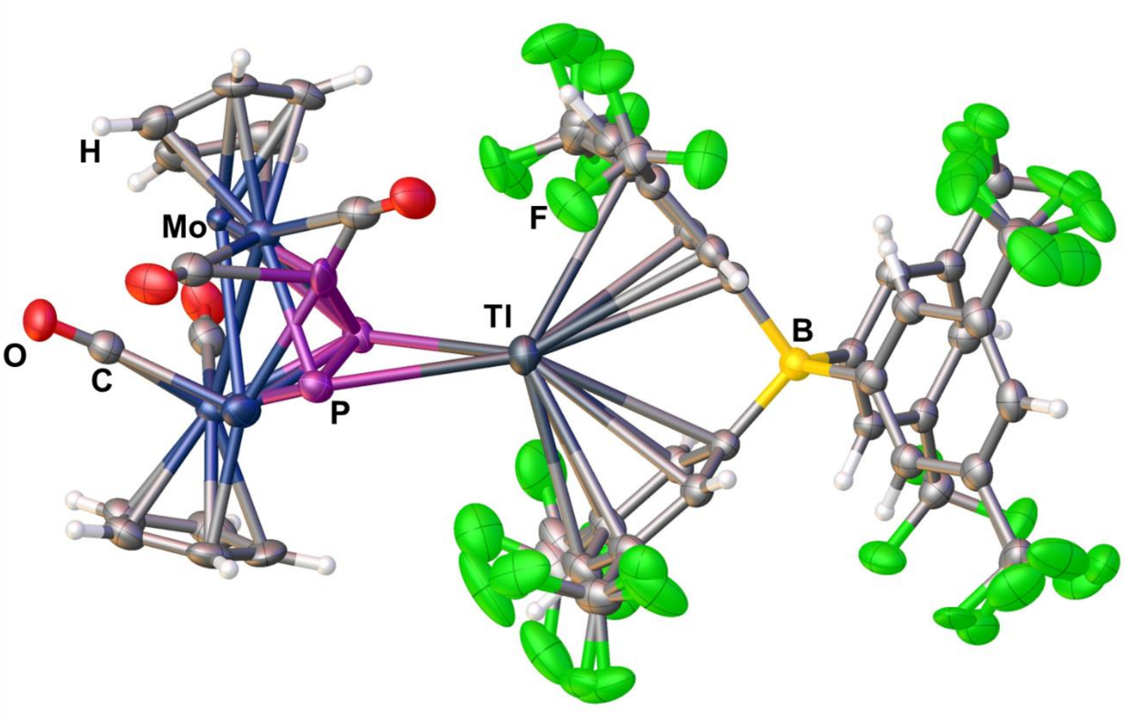

Figure S17: Solid state structure of [**A**]**Tl**. Depicted is the asymmetric unit and ADPs (anisotropic displacement parameters) are drawn at 50 % probability.

[Tl(η^2-**B**)][BArF$_{24}$] ([**B**]**Tl**)

Compound [Tl(η^2-**B**)][BArF$_{24}$] ([**B**]**Tl**) crystallizes from a concentrated solution in *o*-DFB layered with *n*-hexane at room temperature in the triclinic space group $P\bar{1}$ as red blocks. The asymmetric unit contains one molecule **B** and one molecule Tl[BArF$_{24}$]. Disorders within the tetrahedrane unit of **B** (occupancy disorder) and within CF_3 groups of [BArF$_{24}$]$^-$ were treated with adequate restraints.

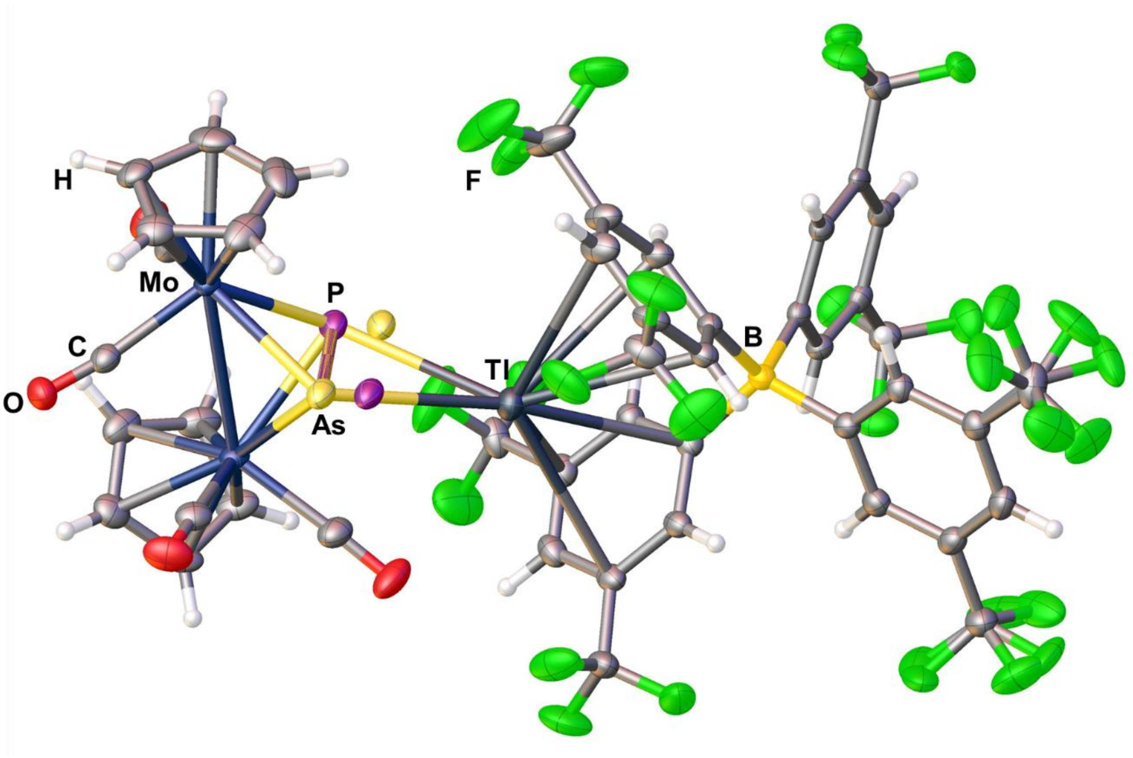

Figure S18: Solid state structure of [**B**]**Tl**. Depicted is the asymmetric unit and ADPs (anisotropic displacement parameters) are drawn at 50 % probability.

[Tl(η^1-**C**)$_2$][BArF$_{24}$] ([**C**]$_2$**Tl**)

Compound [Tl(η^1-**C**)$_2$][BArF$_{24}$] ([**C**]$_2$**Tl**) crystallizes from a concentrated solution in *o*-DFB layered with *n*-hexane at room temperature in the monoclinic space group *C*2/*c* as dark purple blocks. The asymmetric unit contains one molecule **C** and a half molecule Tl[BArF$_{24}$]. Disorders within the CF_3 groups of [BArF$_{24}$]$^-$ were treated with adequate restraints.

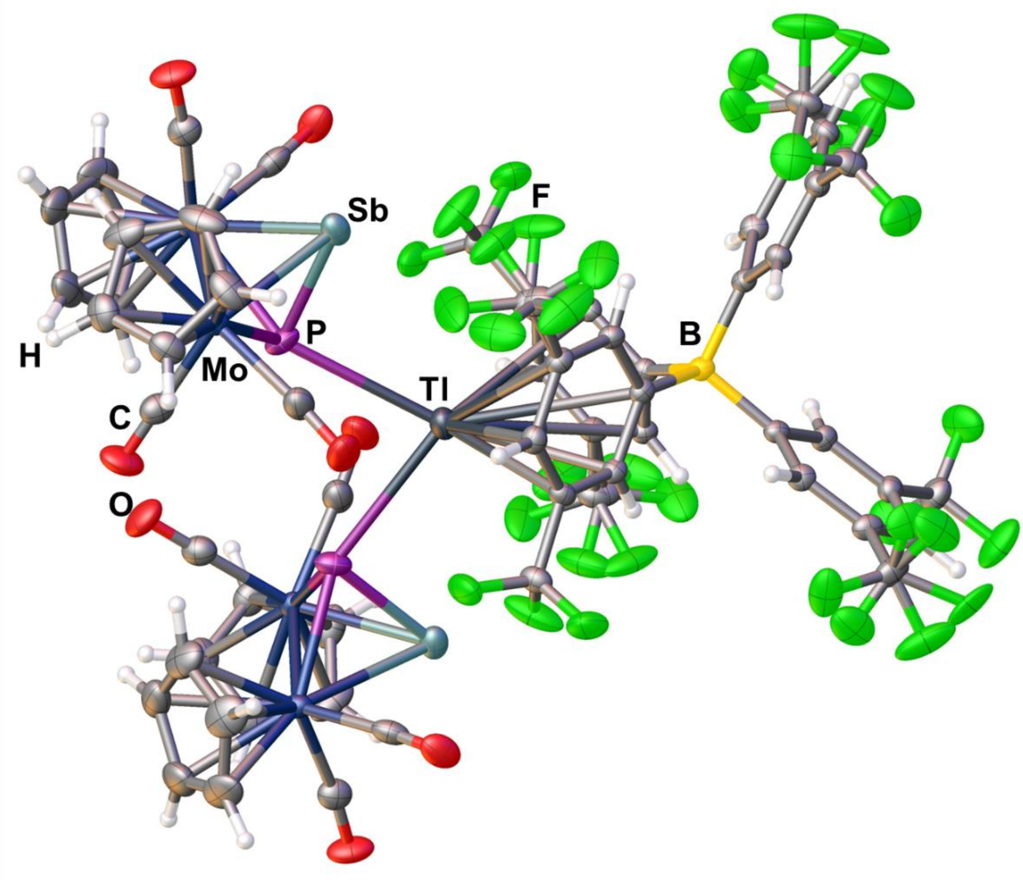

Figure S19: Solid state structure of [**C**]$_2$**Tl.** Depicted are two asymmetric units and ADPs (anisotropic displacement parameters) are drawn at 50 % probability.

[$Tl_2(\eta^2$-**D**$)_3(\mu,\eta^{2:1}$-**D**$)(\mu,\eta^{1:1}$-**D**$)][BArF_{24}]_2$ ([**D**]$_5$**Tl**$_2$)

Compound [$Tl_2(\eta^2$-**D**$)_3(\mu,\eta^{2:1}$-**D**$)(\mu,\eta^{1:1}$-**D**$)][BArF_{24}]_2$ ([**D**]$_5$**Tl**$_2$) crystallizes from a concentrated solution in *o*-DFB layered with *n*-hexane at room temperature in the monoclinic space group $P2_1/c$ as dark red plates. The asymmetric unit contains five molecules **D** and two molecules $Tl[BArF_{24}]$. Disorders within CF_3 groups of $[BArF_{24}]^-$ were treated with adequate restraints.

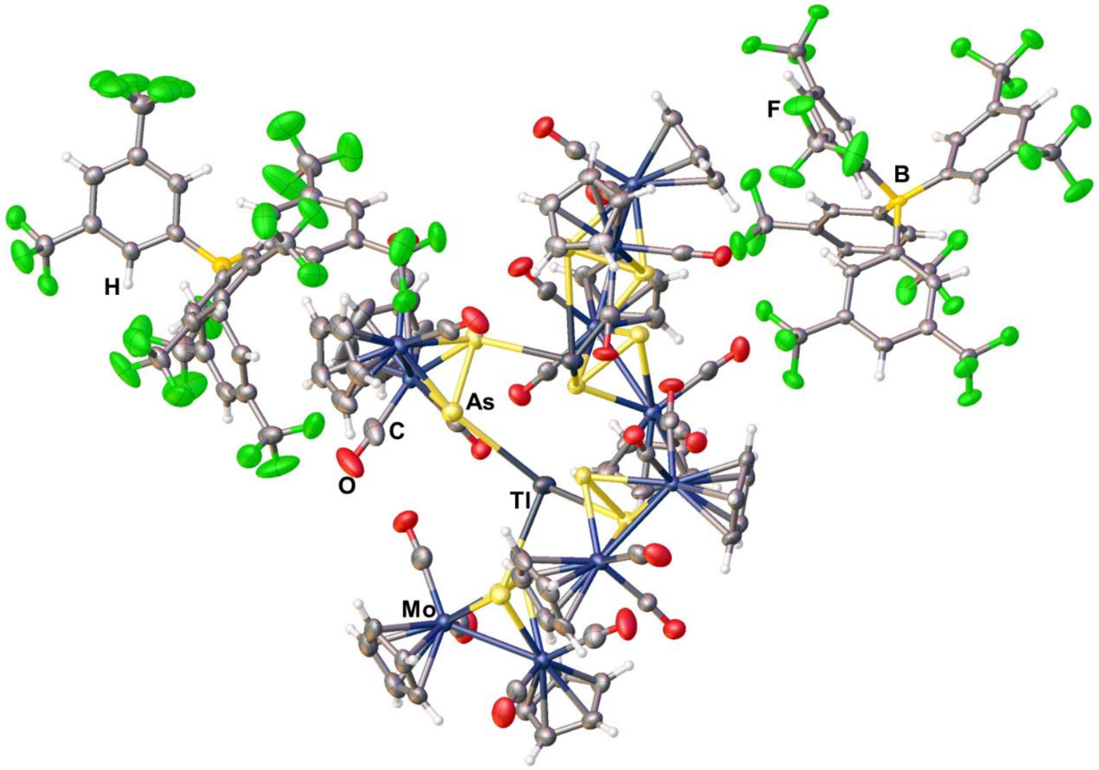

Figure S20: Solid state structure of [**D**]$_5$**Tl**$_2$. Depicted is the asymmetric unit and ADPs (anisotropic displacement parameters) are drawn at 50 % probability.

$[Tl_2(\eta^2\text{-}\mathbf{E})_2(\mu,\eta^{2:1}\text{-}\mathbf{E})_3][BArF_{24}]_2$ (**[E]₅Tl₂**)

Compound $[Tl_2(\eta^2\text{-}\mathbf{E})_2(\mu,\eta^{2:1}\text{-}\mathbf{E})_3][BArF_{24}]_2$ ($\mathbf{[E]_5Tl_2}$) crystallizes from a concentrated solution in *o*-DFB layered with *n*-hexane at room temperature in the monoclinic space group $P2_1/c$ as dark red plates. The asymmetric unit contains five molecules **E** and two molecules $Tl[BArF_{24}]$. Disorders within the tetrahedrane units of **E** and within CF_3 groups of $[BArF_{24}]^-$ were treated with adequate restraints.

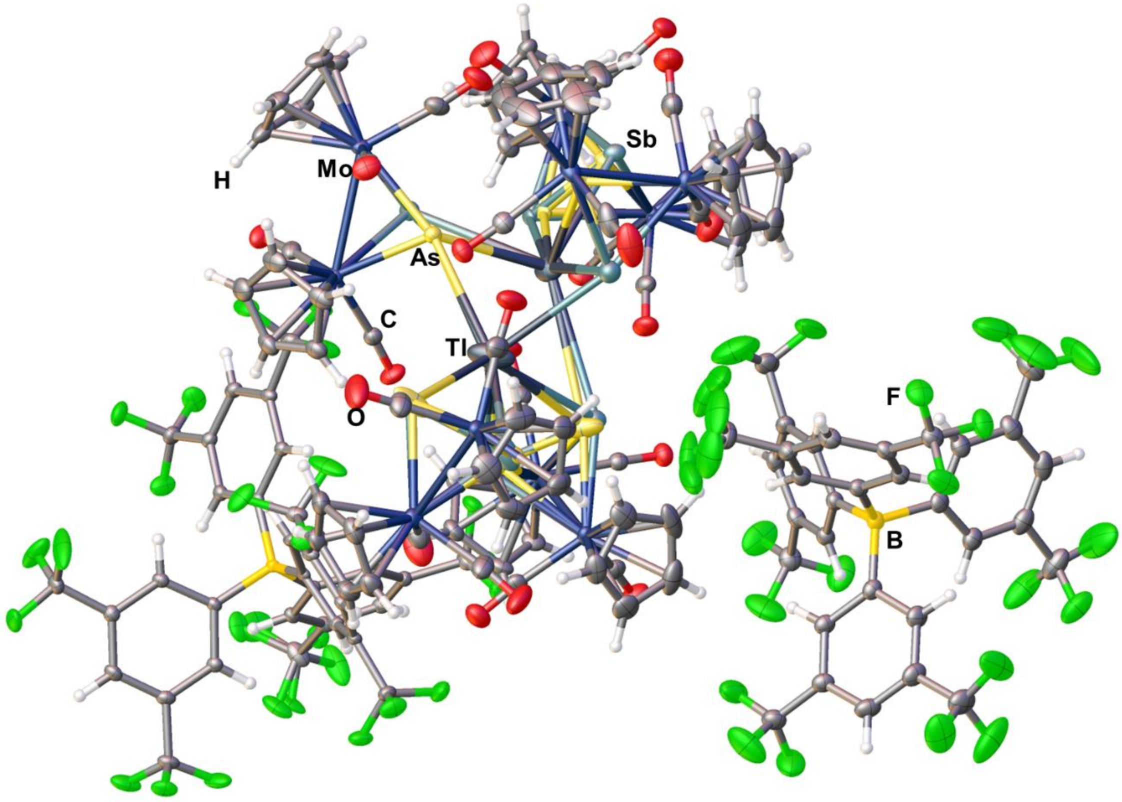

Figure S21: Solid state structure of $\mathbf{[E]_5Tl_2}$. Depicted is the asymmetric unit and ADPs (anisotropic displacement parameters) are drawn at 50 % probability.

$[Tl_2(\eta^2\text{-}\mathbf{F})_3(\mu,\eta^{2:1}\text{-}\mathbf{F})_3][BArF_{24}]_2$ (**[F]$_6$Tl$_2$**)

Compound $[Tl_2(\eta^2\text{-}\mathbf{F})_3(\mu,\eta^{2:1}\text{-}\mathbf{F})_3][BArF_{24}]_2$ (**[F]$_6$Tl$_2$**) crystallizes from a concentrated solution in *o*-DFB layered with *n*-hexane at 4 °C in the triclinic space group $P\bar{1}$ as dark black blocks. The asymmetric unit contains six molecules **F** and two molecules Tl[$BArF_{24}$]. Disorders within the tetrahedrane units of **F** and within CF_3 groups of $[BArF_{24}]^-$ were treated with adequate restraints. The asymmetric unit contained moreover three units of *o*-DFB, which were squeezed applying a solvent mask.

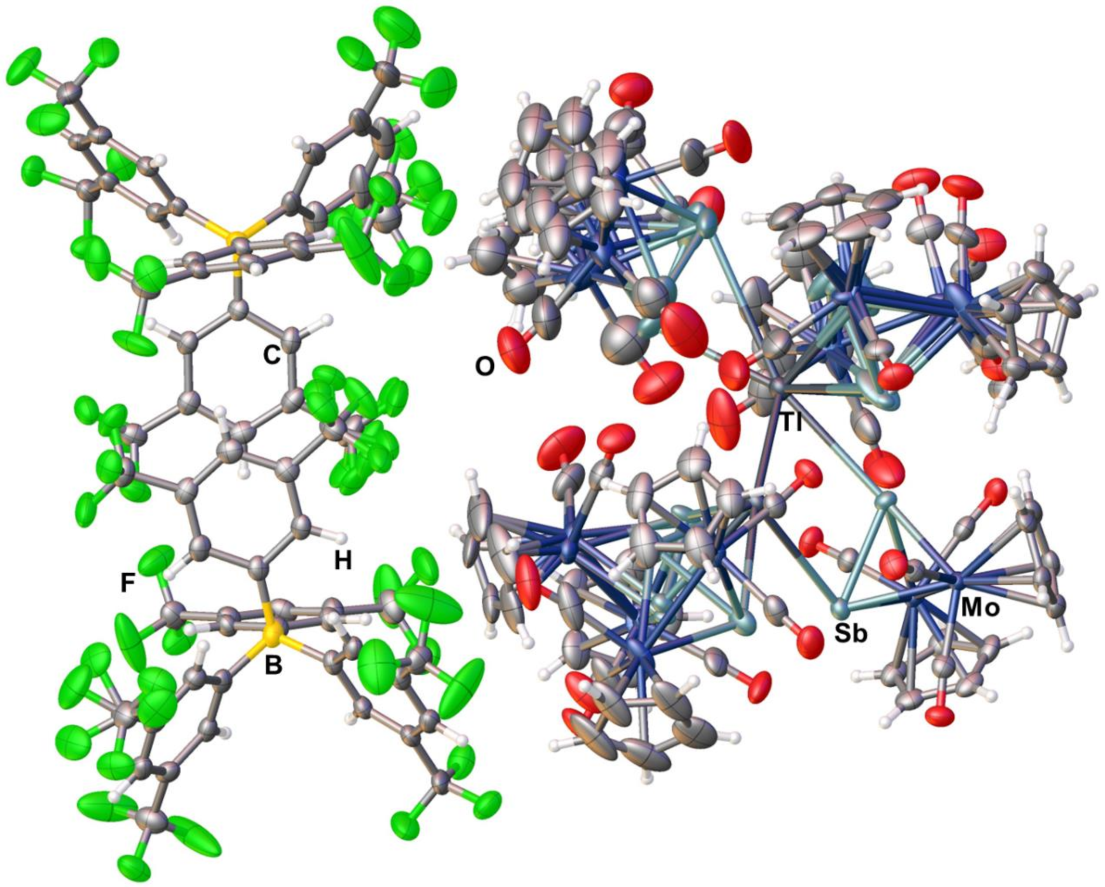

Figure S22: Solid state structure of **[F]$_6$Tl$_2$**. Depicted is the asymmetric unit and ADPs (anisotropic displacement parameters) are drawn at 50 % probability.

Table S1: X-ray crystallographic data of compounds [**A**]**Tl**, [**B**]**Tl** and [**C**]$_2$**Tl**.

Compound	A[Tl]	B[Tl]	C2[Tl]
**Formula**	$C_{46}H_{22}BF_{24}Mo_2O_4P_2Tl$	$C_{23}H_{11}As_{0.5}B_{0.5}F_{12}MoO_2P_{0.5}Tl_{0.5}$	$C_{60}H_{32}BF_{24}Mo_4O_8P_2Sb_2Tl$
ρ_{calc}	2.051	2.109	2.182
μ/mm^{-1}	11.828	12.257	17.967
**Formula Weight**	1563.63	803.79	2241.23
**Colour**	clear red	clear red	clear dark purple
**Shape**	block-shaped	block-shaped	block-shaped
**Size/mm^3**	0.35×0.30×0.23	0.34 × 0.16 × 0.16	0.40×0.19×0.18
***T*/K**	123.00(10)	123.00(10)	123.01(10)
**Crystal System**	triclinic	triclinic	monoclinic
**Space Group**	$P\bar{1}$	$P\bar{1}$	*C*2/*c*
***a*/Å**	14.2024(2)	14.3208(8)	24.6500(3)
***b*/Å**	15.0022(2)	14.9664(11)	16.49250(10)
***c*/Å**	15.2543(2)	15.2825(10)	19.3569(2)
α/°	61.4240(10)	60.842(7)	90
β/°	76.9380(10)	76.792(5)	119.910(2)
γ/°	62.4920(10)	62.244(7)	90
**V/Å^3**	2531.53(7)	2531.3(4)	6821.23(17)
Z	2	4	4
Z'	1	2	0.5
**Wavelength/Å**	1.54184	1.54184	1.54184
**Radiation type**	Cu K_α	Cu K_α	Cu K_α
Θ_{min}/°	3.685	7.462	3.601
Θ_{max}/°	66.648	134.63	66.931
**Measured Refl's.**	41857	24776	28859
**Indep't Refl's**	8863	8889	6042
**Refl's I≥2 *s*(I)**	8840	8889	5962
R_{int}	0.0486	0.0485	0.0454
**Parameters**	925	787	526
**Restraints**	194	73	195
**Largest Peak**	1.778	2.30	1.031
**Deepest Hole**	-1.553	-1.11	-1.559
**GooF**	1.136	1.091	1.088
ωR_2 **(all data)**	0.0939	0.1073	0.0908
ωR_2	0.0938	0.1061	0.0904
R_1 **(all data)**	0.0349	0.0444	0.0336
R_1	0.0349	0.0417	0.0333

Table S2: X-ray crystallographic data of compounds [**D**]$_5$**Tl$_2$**, [**E**]$_5$**Tl$_2$** and [**F**]$_6$**Tl$_2$**.

**Compound**	**[D]$_5$Tl$_2$**	**[E]$_5$Tl$_2$**	**[F]$_6$Tl$_2$**
**Formula**	$C_{134}H_{74}B_2O_{20}F_{48}As_{10}Mo_{10}Tl_2$	$C_{134}H_{74}As_5B_2F_{48}Mo_{10}O_{20}Sb_5Tl_2$	$C_{166}H_{96}B_2F_{54}Mo_{12}O_{24}Sb_{12}Tl_2$
**ρ_{calc}**	2.224	2.308	2.333
**μ/mm^{-1}**	14.090	4.970	4.320
**Formula Weight**	5054.89	5289.04	6543.06
**Colour**	clear dark red	dark red	dark black
**Shape**	plate-shaped	plate-shaped	block-shaped
**Size/mm^3**	0.23×0.15×0.06	0.30×0.19×0.02	0.25×0.18×0.14
***T*/K**	123.00(10)	123.01(10)	122.99(10)
**Crystal System**	monoclinic	monoclinic	triclinic
**Space Group**	$P2_1/c$	$P2_1/c$	$P\bar{1}$
***a*/Å**	36.6827(3)	36.7952(4)	17.6484(3)
***b*/Å**	24.7041(2)	24.7848(3)	23.1895(4)
***c*/Å**	16.80190(10)	16.83740(10)	23.7463(4)
**α/°**	90	90	101.9370(10)
**β/°**	97.3980(10)	97.5690(10)	99.2990(10)
**γ/°**	90	90	94.7920(10)
**V/Å^3**	15099.4(2)	15221.3(3)	9314.1(3)
Z	4	4	2
Z'	1	1	1
**Wavelength/Å**	1.54184	0.71073	0.71073
**Radiation type**	Cu K_α	Mo K_α	Mo K_α
**Θ_{min}/°**	2.162	2.047	2.037
**Θ_{max}/°**	73.772	30.508	25.123
**Measured Refl's.**	150061	268886	104186
**Indep't Refl's**	29116	46450	33195
**Refl's I≥2 *s*(I)**	23419	36942	28182
**R_{int}**	0.0640	0.0488	0.0431
**Parameters**	2063	2161	2625
**Restraints**	36	150	617
**Largest Peak**	1.958	1.426	2.147
**Deepest Hole**	-1.517	-1.366	-2.218
**GooF**	1.054	1.018	1.063
**ωR_2 (all data)**	0.1421	0.0669	0.0893
**ωR_2**	0.1348	0.0624	0.0863
**R_1 (all data)**	0.0653	0.0475	0.0456
**R_1**	0.0512	0.0304	0.0372

5. Computational Details

Within our previous investigations on the reactivity of [{CpMo(CO)$_2$}$_2$(μ,η$^{2:2}$-P$_2$)] (**A**), [{CpMo(CO)$_2$}$_2$(μ,η$^{2:2}$-PAs)] (**B**), [{CpMo(CO)$_2$}$_2$(μ,η$^{2:2}$-PSb)] (**C**), [{CpMo(CO)$_2$}$_2$(μ,η$^{2:2}$-As$_2$)] (**D**), [{CpMo(CO)$_2$}$_2$(μ,η$^{2:2}$-Sb$_2$)] (**F**) towards different coinage metal salts, we already compared the respective energies of the lone-pairs and the σ(E-E') bonds with each other.[5] A summarized energy orbital diagram adapted from the previous publications mentioned above is presented in Figure S22.

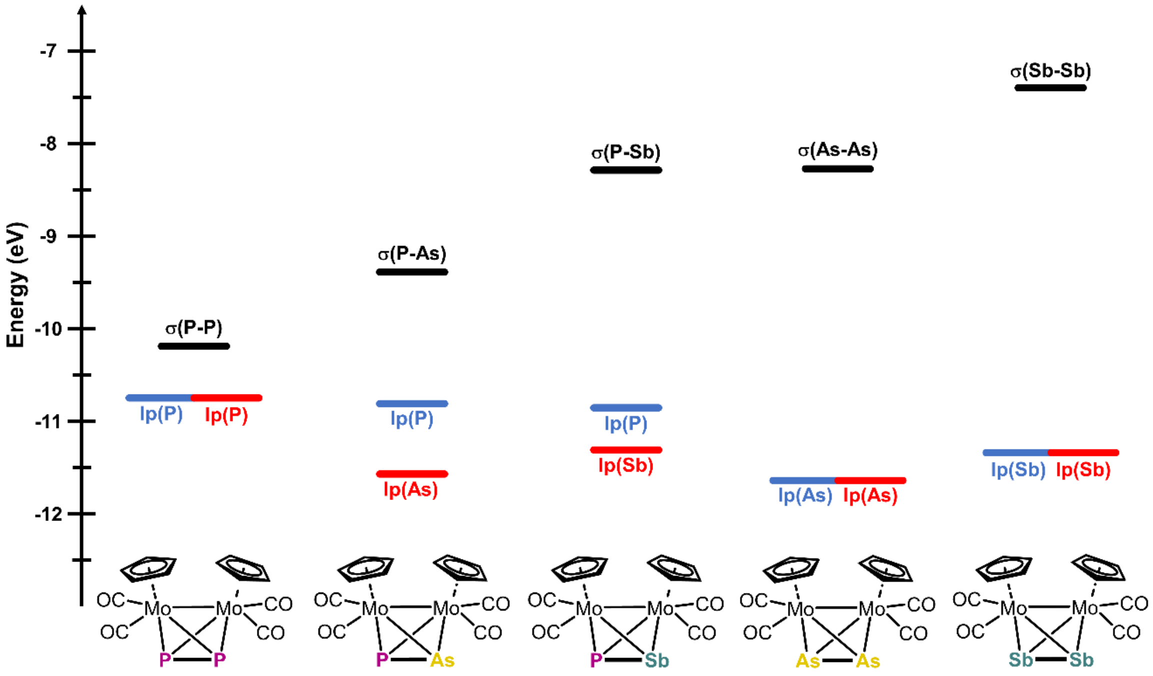

Figure S23: Energy diagram of selected Natural Bond Orbitals (NBOs) for compounds [{CpMo(CO)$_2$}$_2$(μ,η$^{2:2}$-EE')]; adapted from previous publications of the Scheer group.[5]

6. References

[1] Agilent, *CrysAlisPro;* Agilent Technologies Ltd, Yarnton, Oxfordshire, England, **2014**.

[2] O. V. Dolomanov, L. J. Bourhis, R. J. Gildea, J. A. K. Howard, H. Puschmann, *J. Appl. Crystallogr.* **2009**, *42*, 339–341.

[3] G. M. Sheldrick, *Acta Cryst. A* **2015**, *71*, 3–8.

[4] G. M. Sheldrick, *Acta Cryst. C* **2015**, *71*, 3–8.

[5] a) M. Elsayed Moussa, M. Seidl, G. Balázs, M. Hautmann, M. Scheer, *Angew. Chem. Int. Ed.* **2019**, *58*, 12903–12907; b) M. E. Moussa, J. Schiller, E. Peresypkina, M. Seidl, G. Balázs, P. Shelyganov, M. Scheer, *Chem. Eur. J.* **2020**, *26*, 14315–14319; c) P. A. Shelyganov, M. Elsayed Moussa, M. Seidl, M. Scheer, *Angew. Chem. Int. Ed.* **2023**, *62*, e202215650;
